# A comprehensive review on methods for promotion of mechanical features and biodegradation rate in amniotic membrane scaffolds

**DOI:** 10.1007/s10856-021-06570-2

**Published:** 2022-03-10

**Authors:** Raana Sarvari, Peyman Keyhanvar, Samira Agbolaghi, Leila Roshangar, Erfan Bahremani, Neda Keyhanvar, Mehdi Haghdoost, Saeed Heidari Keshel, Afsaneh Taghikhani, Nima Firouzi, Amir Valizadeh, Elham Hamedi, Mohammad Nouri

**Affiliations:** 1 Stem Cell And Regenerative Medicine Institute, Tabriz University of Medical Sciences, Tabriz, Iran; 2 Stem Cell Research Center, Tabriz University of Medical Sciences, Tabriz, Iran; 3 Department of Medical Nanotechnology, School of Advanced Medical Sciences, Tabriz University of Medical Sciences, Tabriz, Iran; 4 Convergence of Knowledge, Technology and Society Network (CKTSN), Universal Scientific Education and Research Network (USERN), Tabriz, Iran; 5 ARTAN1100 Startup Accelerator, Tabriz, Iran; 6 Chemical Engineering Department, Faculty of Engineering, Azarbaijan Shahid Madani University, P.O. BOX: 5375171379 Tabriz, Iran; 7 Alavi Ophthalmological Treatment and Educational Center, Tabriz University of Medical Sciences, Tabriz, Iran; 8 Student Research Committee, Tabriz University of Medical Sciences, Tabriz, Iran; 9 Gene Yakhteh Keyhan (Genik) Company (Ltd), Pharmaceutical Biotechnology Incubator, Tabriz University of Medical Sciences, Tabriz, Iran; 10 Department of Infectious Diseases, Tabriz University of Medical Sciences, Tabriz, Iran; 11 Medical Nanotechnology Research Center, Shahid Beheshti University of Medical Sciences, Tehran, Iran; 12 Department of Chemistry, Tabriz Branch, Islamic Azad University, Tabriz, Iran; 13 Stem Cell and Tissue Engineering Research Laboratory, Chemical Engineering Faculty, Sahand University of Technology, P.O.BOX:51335–1996 Tabriz, Iran; 14 Phil and Penny Knight Campus for Accelerating Scientific Impact, University of Oregon Eugene, OR, 97403 USA; 15 Drug Applied Research Center, Tabriz University of Medical Sciences, Tabriz, Iran; 16 Department of Tissue Engineering and Applied Cell Science, School of Advanced Technologies in Medicine, Shahid Beheshti University of Medical Sciences, Tehran, Iran; 17 Department of Reproductive Biology, Faculty of Advanced Medical Sciences, Tabriz University of Medical Sciences, Tabriz, Iran

## Abstract

Amniotic membrane (AM) is a biological tissue that surrounds the fetus in the mother’s womb. It has pluripotent cells, immune modulators, collagen, cytokines with anti-fibrotic and anti-inflammatory effect, matrix proteins, and growth factors. In spite of the biological characteristics, some results have been released in preventing the adhesion on traumatized surfaces. Application of the AM as a scaffold is limited due to its low biomechanical resistance and rapid biodegradation. Therefore, for using the AM during surgery, its modification by different methods such as cross-linking of the membrane collagen is necessary, because the cross-linking is an effective way to reduce the rate of biodegradation of the biological materials. In addition, their cross-linking is likely an efficient way to increase the tensile properties of the material, so that they can be easily handled or sutured. In this regard, various methods related to cross-linking of the AM subsuming the composite materials, physical cross-linking, and chemical cross-linking with the glutraldehyde, carbodiimide, genipin, aluminum sulfate, etc. are reviewed along with its advantages and disadvantages in the current work.

## Introduction

Different kinds of biomaterials have been applied in the purposeful manufacturing of three dimensionel (3D) scaffolds by numerous fabrication techniques. The biological properties, together with the physical and mechanical characteristics of a biomaterial scaffold, are very prominent, because they could conspicuously affect the growth and function of freshly formed tissue. Perfectly, it is vital for a tissue engineered scaffold to be biodegradable, porous, biocompatible and mechanically competent which affords suitable physical and biological signs for perfect tissue regeneration [1–13]. Biologic scaffolds with the natural extracellular matrix (ECM) are able to regulate the tissue curing and remodeling routes for functional retrieval of the damaged tissue. Different from synthetic scaffolds, the biologic scaffolds are biologically active, biodegradable and also manipulated by the bioactive molecules and ECM proteins for curing and repairing the tissue. The human amniotic membrane (HAM) has been used in different clinical applications, particularly those related to the ocular surface renewal and wound managing. It contains unique biological and mechanical properties, which makes it favorable among other well-known scaffolding materials. The HAM patching can support the healing processes in ocular surface disorders [14, 15], infarcted hearts [16–18], liver fibrosis [19] and skin wounds [20, 21]; because it has the anti-inflammatory [22], anti-fibrotic, low immunogenic features and it also excretes the soluble factors critical for cell growth and differentiation [19, 23–25]. The HAM efficiently adheres to the wound and retains a humid microenvironment at the injury site. Not only the HAM is relatively thin, but also it is sufficiently strong and elastic making it a proper biomaterial candidate for tissue engineering applications [26]. Despite these properties, the HAM dissolve in the body progressively. Spoerl et al. [27] have reported that both fresh and cryopreserved HAM can entirely dissolve in 1 week, probably because of internal enzymatic degradation of the HAM matrix.

In case of the corneal ulcer, Stevens-Johnson’s syndrome or chemical burn that could origin from an intense corneal inflammation and extreme proteolysis, in a rare instance the transplanted HAM may dissolve and lead to surgical defeat [28]. In case of corneal tissue engineering, the HAM as a cell culture scaffold, should be augmented in order to improve the material stability against enzymatic cleavage. Since the architecture framework of the AM has been established from collagen [29], thus it can undertake the cross-linking readily, a process that create bonds between the collagen chains of the bridge. The cross-linking approach that stabilizes the AM collagen are divided into two chemical and physical methods such as glutaraldehyde cross-linking [27, 29], and irradiation with electron beam [29] or gamma-ray [29, 30] respectively. The physical method does not cause a potential damage, but have difficulty in balancing the cross-linking density of the collagens [27, 31]. Hence, in this review we will focus on cross-linking methods regarding the type IV collagen of the HAM that affect the mechanical properties which will end in increased durability of the collagenous materials.

## Anatomy of the amniotic membrane

**Fig. 1:**
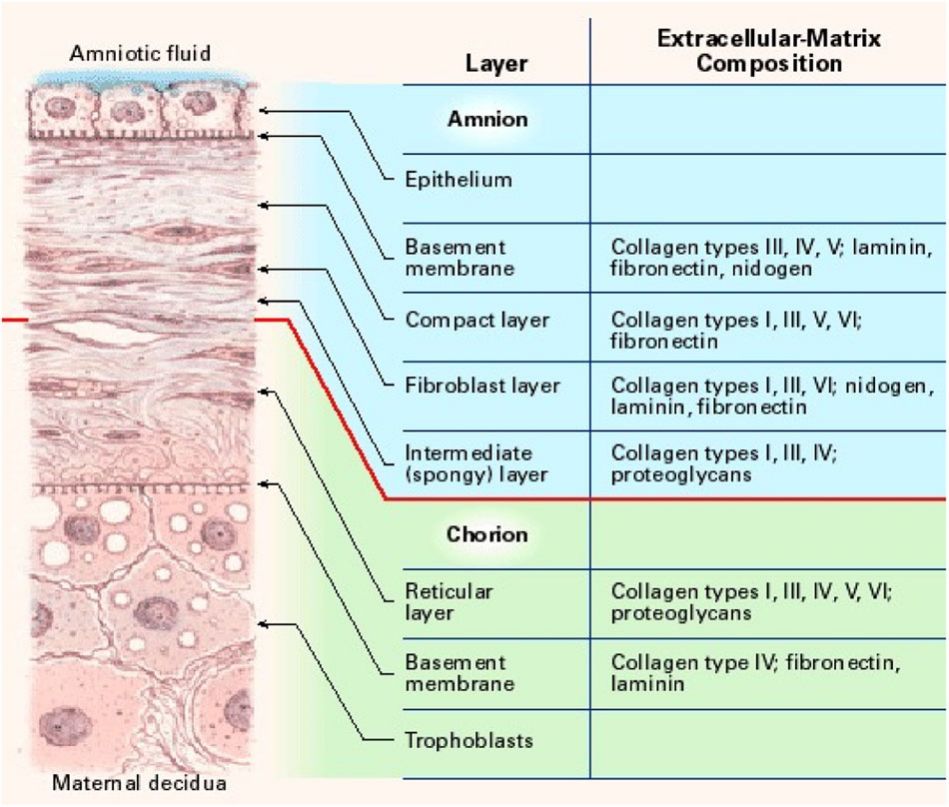
Schematic structure of the fetal membrane [23, 32]

The AM progress from extra-embryonic tissue and contain a maternal constituent (the deciduas) and a fetal precursor (the chorionic plate). They are attached by the chorionic villi and linked to the cytotrophoblastic shell of the chorionic sac to the decidua basalis. The fetus is detaches from the endometrium by fetal constituent, which contains the chorionic and amniotic fetal membranes. The amniochorionic membrane forms the outer layer of the sac that surrounds the fetus, while the AM is the innermost layer of the sac. The AM involves an avascular stroma, a dense basement membrane, and a monolayer of epithelium (Fig. 1). It contains no nerves or blood vessels and all the nutrients that AM needs are provided by the propagation outside of the amniotic fluid and also from the emphasizing decidua. The innermost layer, which is also the nearest to the fetus is called the amniotic epithelium that involves a single layer of the cells arranged on the basement membrane regularly. The basement membrane is one of the densest membranes found in between all human tissues. The structural integrity of this important membrane is proved by its backing provided by the basement membrane to the fetus all over pregnancy. The condensed layer of the stromal matrix adjoining to the basement membrane forms the main fibrous skeleton of the AM. The collagen abundant layer is discharged by the mesenchymal cells of the fibroblast layer. The predominant collagens types I and III form parallel packs that keep the mechanical integrity of the AM. The epithelial basement membrane is linked to the interstitial collagens (types I and III) by the aim of collagens type V and VI by forming a filamentary link. The middle layer (spongy layer) of the stromal matrix is also joined to the chorionic membrane. Its plentiful glycoproteins and proteoglycans yields a spongy structure in histologic devising, and it possess mostly type III collagen as non-fibrillar latticework [32]. The spongy layer is joined to the chorionic membrane loosely; hence, the AM is separated easily by means of straight dissection from the chorion [23].

## Mechanical properties and mechanisms

The AM possesses numerous characteristics that make it appropriate for being used in tissue engineering [23]. When considering the epithelial layer of the AM, the cells are remarkably similar to the stem cells. They are pluripotent and are independent from a feeder layer during their cultivation and have the potential to differentiate into all three germ layers [23]. Other important characteristics of the AM such as anti-fibrotic, anti-tumorigenic, low immunogenicity, antimicrobial, anti-inflammatory, anti-scaring, and valuable mechanical properties are also critical [23]. However, there exist some challenges for the application of AM in tissue engineering. As an instance, it has a thin structure and shows technical restrictions when suturing. So, it has been recommended to use the adhesives for suturing as a substitute [33]. Besides, the AM displays a viscoelastic mechanical reaction [23]. In most tissues, the viscoelasticity is essential for scaffolding, e.g., the rigid scaffolds of the blood vessels that may boost hyperplasia and blocking [34]. Nevertheless, the rigidity of AM is more valid for most protocols in tissue engineering [35]. It has been recommended that this may be correlated to the collagen content, though there are opposing studies which show that the content of amnion collagen decreases with Pregnancy age [36]. Furthermore, it is also suggested that elastin of the fetal amnion provides the molecular origin related to the elasticity of AM [37].

The location of the AM may be different, whether taken from distal or adjacent placental disk. It has been established that adjacent human samples of AM are stronger and thicker having weaker optical properties ratio compared to distal samples [38]. Also, AM may be applied either fresh or modified over altered preservation methods, for example, freezing, lyophilization or cryopreservation in surgical procedures [39]. Cryopreservation ratio to freezing, seeks to reach very low temperature without causing further damage to freezing ice. Also, better preservation of growth factors has been reported for cryopreservation compared to freezing [39]. When comparing the fresh and cryopreserved AM, according to reports, the epithelial cells were less viable and had poor proliferative ability in cryopreservation method. Morphological differences were not identified between cryopreserved and fresh AM [40]. It has been reported that the risk of transmitted infections may decreases when preserving and sterilizing the AM by para acetic acid, gamma irradiation, and/or trehalose [39]. Different growth factors such as hepatocyte growth factor (HGF), epidermal growth factor (EGF), basic fibroblast growth factor (BFGF), transforming growth factor b (TGFb), and platelet-derived growth factor (PDGF) are secreted by the AM [41, 42]. EGF is mainly found in the amniotic epithelium and is a powerful mitogen for the epithelial cells growth and its high level of expression may elucidate for the ocular surface improved wound healing [25, 39]. PDGF result in cellular responses including migration, proliferation, survival and the deposition of ECM along with the tissue remodeling factors [43]. Koizumi et al. [25] reported that keratinocyte growth factor (KGF), and HGF secreted from the amniotic epithelium are also created by mesenchymal cells such as corneal stroma fibroblasts. Growth factors of the AM epithelium may affect corneal wound healing via paracrine action [44, 45]. It is also suggested that re-epithelialization of ocular surface may be enhanced by KGF and HGF which is secreted by the amniotic epithelium will help AM transplantation (AMT). Moreover, AM has also anti-inflammatory effect [14, 46, 47]. Human limbal epitelial cell (LEC) cultured on the AM stromal matrix significantly suppresses the expression of IL-1a and IL-1b, even when confronted by bacterial derived lipopolysaccharides [47]. In a study, in which, after keratectomy of phototherapeutic, the corneas of rabbits were shielded by human AM, results indicated that, the apoptosis of polymorph nuclear neutrophils significantly decreased the acute inflammatory reaction [48]. This finding was also reinforced in patients with severe burns where AM trapped CD201 lymphocytes and exhibited cell death [49]. It has been demonstrated that after inoculation of the rat cornea with herpes simplex virus type 1 for inducing necrotizing keratitis, covering cornea by preserved human AM decreased inflammation [50]. The limbal stem cell deficiency (LSCD) can happen by chronic inflammation in the limbal region. Moreover, In the LSCD treatment, inflammation can negatively affect integration of auto-grafts of transplanted conjunctival-limbal [51]. Therefore, AM may have significant benefits due to its anti-inflammatory characteristic. Various factors contribute in the antifibrotic effect of the AM [52, 53]. Tseng et al. [53, 54] have reported that signaling downregulation of the TGFb is responsible for fibroblasts activation during wound healing.

## Human amniotic epithelium (HAE) as a source of stem cells (SC)

Human placentas, which are normally discarded after delivery constituted valuable sources of maternal and fetal cells, exhibit the superior plasticity [55–58]. Huge attention has been forwarded to the human amniotic epithelial cells (hAECs) as a source of progenitor cells of fetal origin with no ethical issue involvement. Some studies manifested that the amniotic epithelial cells from different species such as rat, sheep, and human possess the combined qualities of both embryonic and adult stem cells and resume a significant plasticity [59–63]. The HAECs have the trilineage differentiation ability in vitro and express markers of both mesenchymal and embryonic stem cells (ESCs) [57, 60, 63, 64]. In contrast to ESCs, the hAECs represented a stable nontumorigenic phenotype, evidenced by several long-term in vivo transplantation experiments [57, 59, 60]. In addition, the fetal origin may provide the hAECs with not only the fetus-maternal immunotolerance but also an immunomodulatory property, thereby supporting the application safety of hAECs in allotransplantation [65–67]. All above-mentioned features make the hAECs a promising and noncontroversial source of progenitor cells for the wide application in cell transplantation and regenerative medicine. In a recent research, the in vitro and in vivo osteogenic ability of amniotic epithelial cells was depicted in distinct studies showing that the amniotic epithelial cells may be an appropriate source of progenitor cells for bone tissue engineering [58, 61, 64, 68]. In contrast, further systemic investigations comparing the regenerative properties of hAECs with other sources of stem cells are required before the feasibility of hAECs in the bone tissue engineering [64, 68].

## Applications

Native hAM has been applied as a scaffold for TE and regeneration in different medical fields. Some reviews describe its use as a grafting material [69] in oral and periodontal surgeries [70], cartilage damage [71], lower extremity repair [72], healing of chronic wounds and ulcers [73] as a biomaterial in urology [74, 75] in gynecology [76], as of patch for cardiac surgery [77] and as a treatment of ocular surface pathologies [78]. The hAM is an outstanding allogeneic graft material. However, to improve the variability of its mechanical properties and the variation of biological performance, some works and patents have discussed employing complementary scaffolding techniques with hAM inclusion, as summarized in Table 1, with the aim of composite creation by the biomaterials, which enhance the hAM scaffold efficacy for different applications [79].

**Table 1:** Application of hAM composites and commercial products [80]

Application	Form of hAM	Membrane material	In vitro test	In vivo test	Reference
Wound healing and tissue regeneration	hAM lyophilized and pulverized	Scaffold with a synthetic polymer and a natural polymer	Keratinocytes and fibroblast	Skin model nude mice and Yorkshire pigs	[81]
	hAM solubilized	Hyaluronic acid hydrogel		Mice	[82]
Reconstructive urology	hAM frozen	Electrospun poly‐(L‐lactide‐co‐E‐caprolactone)	Mesenchymal stem cells	Wistar rats	[83]
Artificial cornea material ‐ Keratoprosthetic	hAM decellularized	Polyvinyl alcohol (PVA)	*–*	Rabbit corneal epithelial cells	[84]
Aligned tissue regeneration	hAM decellularized	Electrospun fibers of PLGA	–	Skeletal muscle cells	[26]
Ocular surface reconstruction	*Amnioguard* (bio‐tissue)	N/A only native hAM	Clinical test 11 patients		[85]
Neuropatical corneal pain	*ProKera* ®(bio‐tissue)	N/A only native hAM	Clinical test 9 patients		[86]
Ocular surface disorders	*AmnioClip‐plus (DGFG)*	N/A only native hAM	Clinical test 7 patients		[87]
Premature rupture fetal membrane	hAM cell‐free scaffold	Polyester urethane scaffold used as a comparison	‐	Rabbit model	[88]
Vascular graft	Rolled hAM	N/A only native hAM	–	Rabbit model	[89]
Tendon regeneration	hAM pulverized	Collagen‐glycosaminoglycan Hyaluronic acid	Tenocytes	–	[90]
Skin regeneration	hAM decellularized	Nanofibrous silk fibroin	Adipose tissue‐derived mesenchymal stem cells	–	[91]

## Modification of AM

The cells are affected by underlying substrate topography, and it has been presented that physical signs control cell migration, morphology, and embryonic development [92]. Using photolithography, studies reported that, surfaces having single 1–5 mm tall bulge was enough to decrease the migration rate of the fibroblast and baby hamster kidney cell types selectively, but not neutrophils [93]. Microarray Analysis for cultured cells on substrates having hexagonal pits in comparison with flat surfaces revealed the significant gene expression changes which were associated with the extracellular matrix protein production and cell cycle regulation [94]. Obtained results show clearly that how small topographies can have a very important impact on cells/tissues regulation, development and homeostasis. It is recognized that structural changes in the molecules constituting the matrix will result in cell signaling alteration [95]. Collagen undergoes many post-translational modifications that are important for its mechanical and structural properties and disruption of these processes will end in cellular dysfunction. Collagen molecules will self-assemble into fibrils after cleavage of the C and N pro-peptides and covalent cross-links formation are the final steps in the collagen formation [96]. Optimal collagen cross-linking is critical for its binding to the receptors; nevertheless, it is also essential for the regulation of growth factors along with the extracellular matrix mechanical characteristics [97]. Early studies have shown that, program of osteogenic is weakened by collagen cross-linking inhibition in the pre-osteoblast cell line. [98]. Moreover, harming the collagen cross-linking is duo to exposure of cryptic nucleation sites, which will result in enhanced mineralization [99]. Inadequate collagen cross-linking makes it more prone to proteolytic degradation [100]. Collagen nanofibers as a vital structural component of the AM, undergo substantial degradation after exposing to endogenous collagenases. The collagenase activity enhancement in many diseases affect the cornea and may thus lead to faster degradation of AMT [101]. Spoerl et al. [102] demonstrated that the important cause of early AM detachment during corneal healing may be due to the insufficient biological stability of AM transplantation. Knowing that, Strategies for enhancement of the AM molecular bio stability is necessary. Subsequently, it is desired that the AM collagen assists as a limbal stem cell niche and nnumerous researchers have attempted to modify it in a cross-linked molecular biopolymer chain. Various cross-linking approaches have been used to enhance AM stability for LEC cultures, including [31, 102–106], glutaraldehyde- [27, 29, 107, 108], radiation- [105], Al_2_(SO_4_)_3_- [109] and photo- crosslinking [110].

### Chemical cross-link

Here are some chemical cross-linking approaches reported for hAM to improve its properties such as mechanical properties, biodegradation rate without affecting the immunophenotype, viability and proliferation of cells cultured on cross-linked hAM [27, 31, 79, 102, 106, 110, 111].

#### Glutaraldehyde (GTA)

**Fig. 2:**
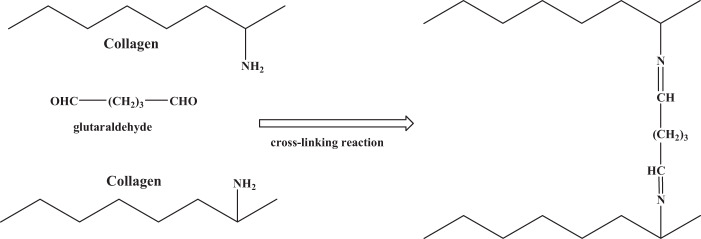
Cross-linking mechanism of AM collagen with GTA

One of the well-known chemical techniques for cross-linking the AM is the usage of glutaraldehyde (GTA). The GTA is a highly functional and effective cross-linking substrate used to stabilize the collagen biomaterials. Cross-linking mechanism of AM collagen with GTA is illustrated in Fig. 2.

**Fig. 3:**
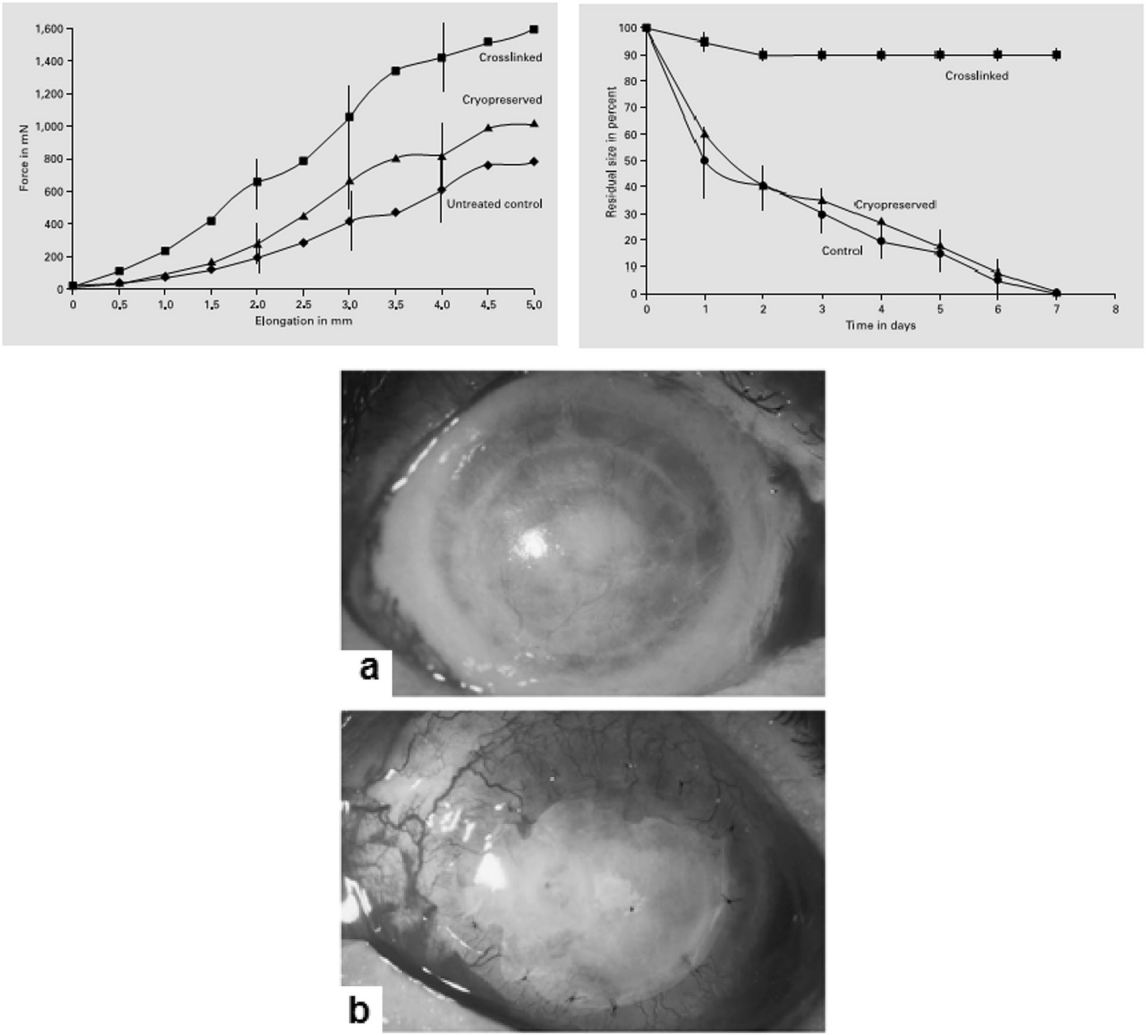
Force-elongation curves of treated and control amniotic membranes (top); the smooth, TAM cross-linked with glutaraldehyde after 35 days (bottom), (**a**); the cryopreserved amnion, 2 days after TAM with partial loss of transparence and dissolution (bottom), (**b**) [27]

Spoerl et al. [27] reported the cross-linking of the by GTA for AMT. They attempted to stabilize the collagen of the AM by cross-linking using glutaraldehyde for its transplantation. According to Table 2, the amnion cross-linked by GTA was significantly increased (76.8%) compared to the cryopreserved amnion and 175% versus fresh amnion. The membranes treated with the glutaraldehyde were resistant to enzymatic digestion, while the fresh and cryopreserved amnions were almost dissolved at day 7. The cross-linked membrane grafted to the patients was maintained within 90 days with no signs of membrane dissolution and showed desired transparency. AMT Clinical data with cross-linked and cryopreserved amnion has been shown in Table 2. Moreover, the force-elongation curves and images of AMT are depicted in Fig. 3(top and bottom), respectively [27].

**Table 2:** AMT Clinical data with cross-linked and cryopreserved amnion [27]

Case	Amnion type	Disease	Time of AMT removal in days	Status of AMT	Therapeutic result
A1	cryopreserved	Dry eye	22	I	+
A2	cryopreserved	Bacterial ulcer	17	P	+
A3	cryopreserved	Bacterial ulcer	2	P	(+)
A4	cryopreserved	Rheumatic ulcer	9	C	–
A5	cryopreserved	Rheumatic ulcer	15	P	–
B1	cross-linked	Neurototrophic ulcer	90	I	+
B2	cross-linked	Rheumatic ulcer	44	I	+
B3	cross-linked	Peters anomaly	74	I	+
B4	cross-linked	Fuchs dystrophy	59 s	I	+
B5	cross-linked	Bacterial ulcer	18	I	+
B6	cross-linked	Lyell syndrome	35	I	+
B7	cross-linked	Chemical burn	49	I	(+)
B8	cross-linked	Bacterial ulcer	12	I	+

*I* Intact, *P* Partially dissolved, *C* Completely dissolved.

**Fig. 4:**
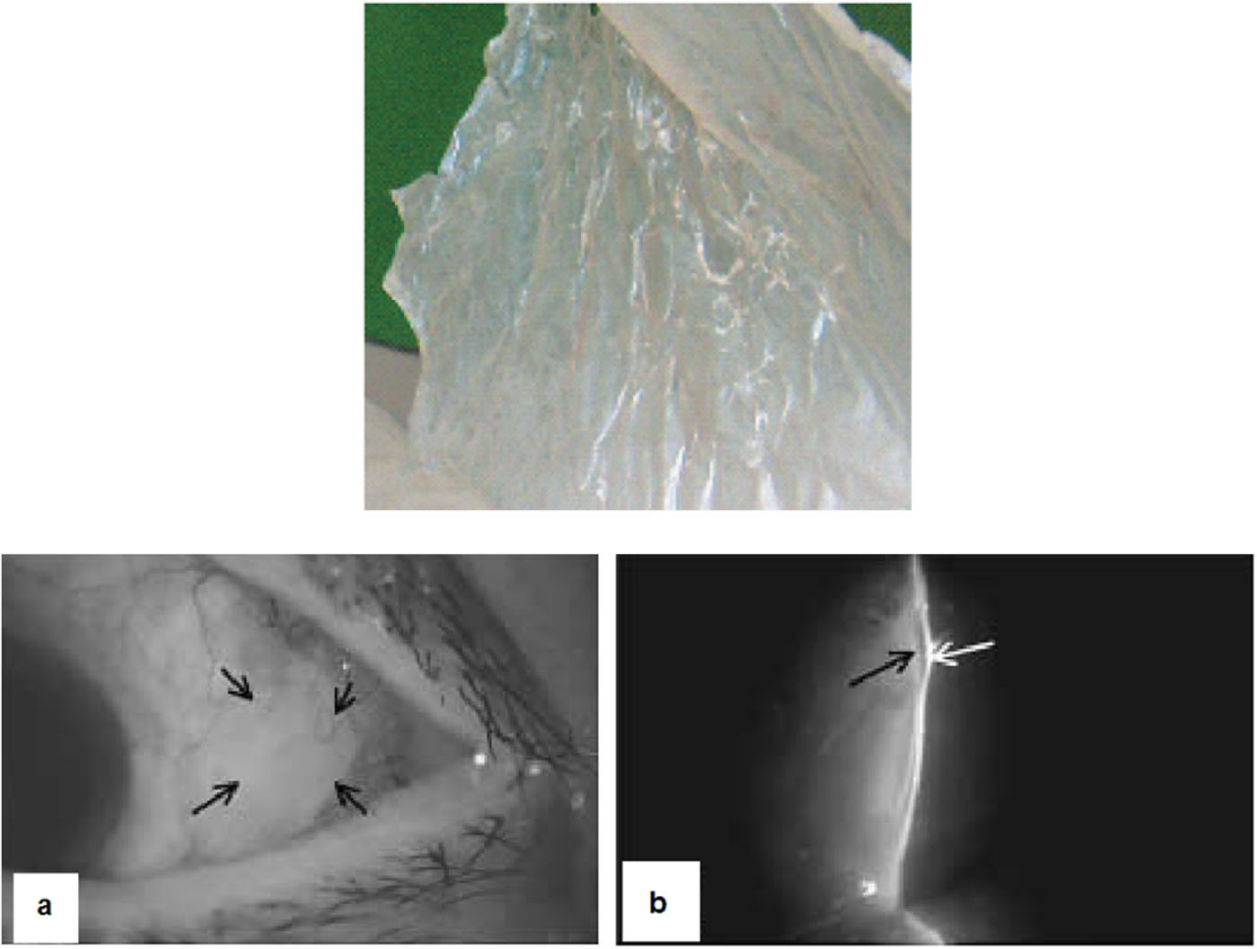
Dried amniotic membrane (Hyper-dry-amnion) was developed by far-infrared rays and microwave, in addition to γ-sterilization (top); 14 days post operation, cross-linked AM was secured under the conjunctival bleb (black arrows) (bottom), (**a**); Regenerative conjunctival epithelium layer (white arrow), was seen over the implanted AM (black arrow) (bottom), (**b**) [116]

On the other side, the chemical modification of AM by GTA possibly reduces the immunity level, especially when the cross-linking reaches a high level [112]. Different studies have illustrated that the use of GTA as a cross linker is not recommended since it has a toxic nature [54, 113, 114]. Kitagawa et al. [115] reported that a patient experienced the trabeculectomy with mitomycin C in an eye. A large conjunctival defect with water leakage suddenly advanced 2 days post intervention. The patient has endured extraction of extracapsular cataract and 5 previous trabeculectomy procedures in eye. AM dried by a new method (Hyper-dry) (Fig. 4(top)), was inserted under the conjunctival bleb via the site of conjunctival defect and glue (2-octyl cyanoacrylate) following cross-linking by glutaraldehyde. It was used in adjacent area inside the conjunctival defect edging for securing the membrane of amniotic to the conjunctival. The naked surface of the transplanted AM was steadily enclosed with the epithelia of conjunctival and the defect was sealed completely 14 days later (Fig. 4(bottom, a) and (bottom, b)). The dried, cross-linked AM did not resolve after the 24-month [116].

**Fig. 5:**
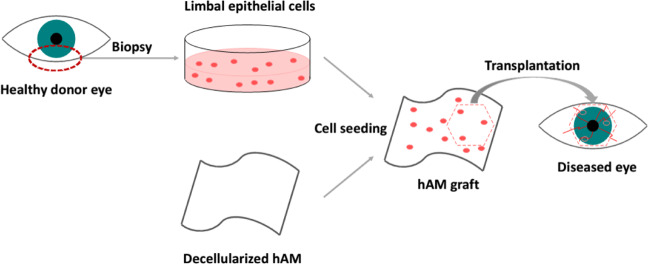
Transplantation process of autologous limbal epithelial cells [79]

The first use of AMT to regenerate corneal and conjunctival levels goes back to the 1940s when dried amnion was used by Symons and Sorsby [117]. Recently, there is a great interest in AMT because it has been shown that corneal epithelium can regenerate following cryopreserved amnion transplantation in spite of losing limbal stem cells when transplanted in rabbits [118]. Rabbit conjunctival epithelium can transdifferentiate when culturing on the basement membrane of the amniotic epithelium [102, 119]. Figure 5 illustrates the transplantation process of the limbal epithelial cells using hAM as a carrier membrane [79].

**Fig. 6:**
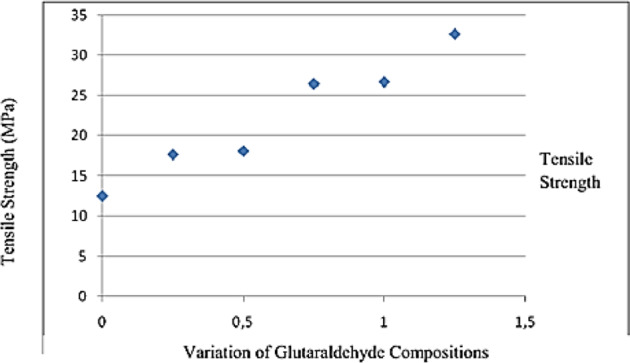
The relationship between glutaraldehyde concentration and tensile strength [121]

A main AMT problem is its quite short life shelf with early removal of the amniotic membrane due to tissue rupture in the suture or progressive dissolution of the membrane in some cases due to epithelial and stromal defects. Hence, it frequently requires a long-time patching than 1–2 month to complete a surface reconstruction [120]. Mostly, the fresh amnion dissolve within 1 week, while the cryopreserved amnion can usually be utilized for long weeks since it is stored in the desired conditions without repeated thawing, especially when covered with a large bandage contact lens. However, in specific disease cases, which have not yet been clearly elucidated, the cryopreserved amnion dissolves after 2–3 weeks. This may end in several consecutive amniotic grafts should be performed [102]. Dian Marta Rizkawati et al. [121] studied the effect of GTA on the HAM cross-linking. For this purpose, the HAM-GTA wound dressing was made by immersing the HAM in the solution of different compositions of GTA with the percentages of 1.25%, 1%, 0.75%, 0.5%, and 0.25%. According to the results of the 26.67 MPa tensile test, the best glutaraldehyde concentration for wound dressing was 1%. The cytotoxicity assay and histopathology anatomy tests revealed that the percentage of the viable cells (113.483%) ended in 100% wound re-epithelialization on the mice’s skins. The tensile strength of the HAM may be a result of an addition of 0.25% and 1.25% GTA in combination (Fig. 6). Based on Vogel’s research in 1987, the final tensile strength of human skin on different spots ranged in 5–32 MPa and the result of increasing GTA to AM at concentration range of 0.25% to 1% was appropriate [27, 122]. The GTA greatly enhances the enzymatic resistance and biomechanical strength of the amnion [27].

According to Table 3, the percentages of viable cells are 116.629, 118.938, 114.382, 119.169, 113.483 and 115.012% for samples A, B, C, D, E and F, respectively. The live cells percentages above 100% in whole sample displayed that the HAM and GTA are used as the raw materials for the construction of HAM-GTA, which is safe for wound dressings because of no toxicity to fibroblasts. A material is also entitled as toxic if the percentage of cells that live is less than 50% [123].

**Table 3:** Percentage of living cells [121]

Sample	Variation of glutaraldehyde addition concentrations	Live cells%
A	0%	116.629
B	0.25%	118.938
C	0.5%	114.382
D	0.75%	119.169
E	1%	113.483
F	1.25%	115.012

The GTA, which is known as a zero-length cross-linker that joins molecules together, may form covalent bonds (Schiff bases) [101, 124, 125]. The cross-links between the polypeptide chains will result in a more rigid network structure. While chemical modification with GTA may have a toxicity risk [126, 127] it is also found to be clinically acceptable for the implantation [128]. The glycine was found to quench the aldehyde groups offered in the cross-linked biomaterials for reducing the cytotoxicity of compounds [129]. In another study, the results indicated that the viability of human retinal pigment epithelial cells for GTA cross-linked gelatin samples with glycine was significantly increased in comparison to equivalents without glycine treatment [130]. The AM materials also underwent the GTA cross-linking in aqueous glycine solution to cover residual aldehyde groups [107].

**Fig. 7:**
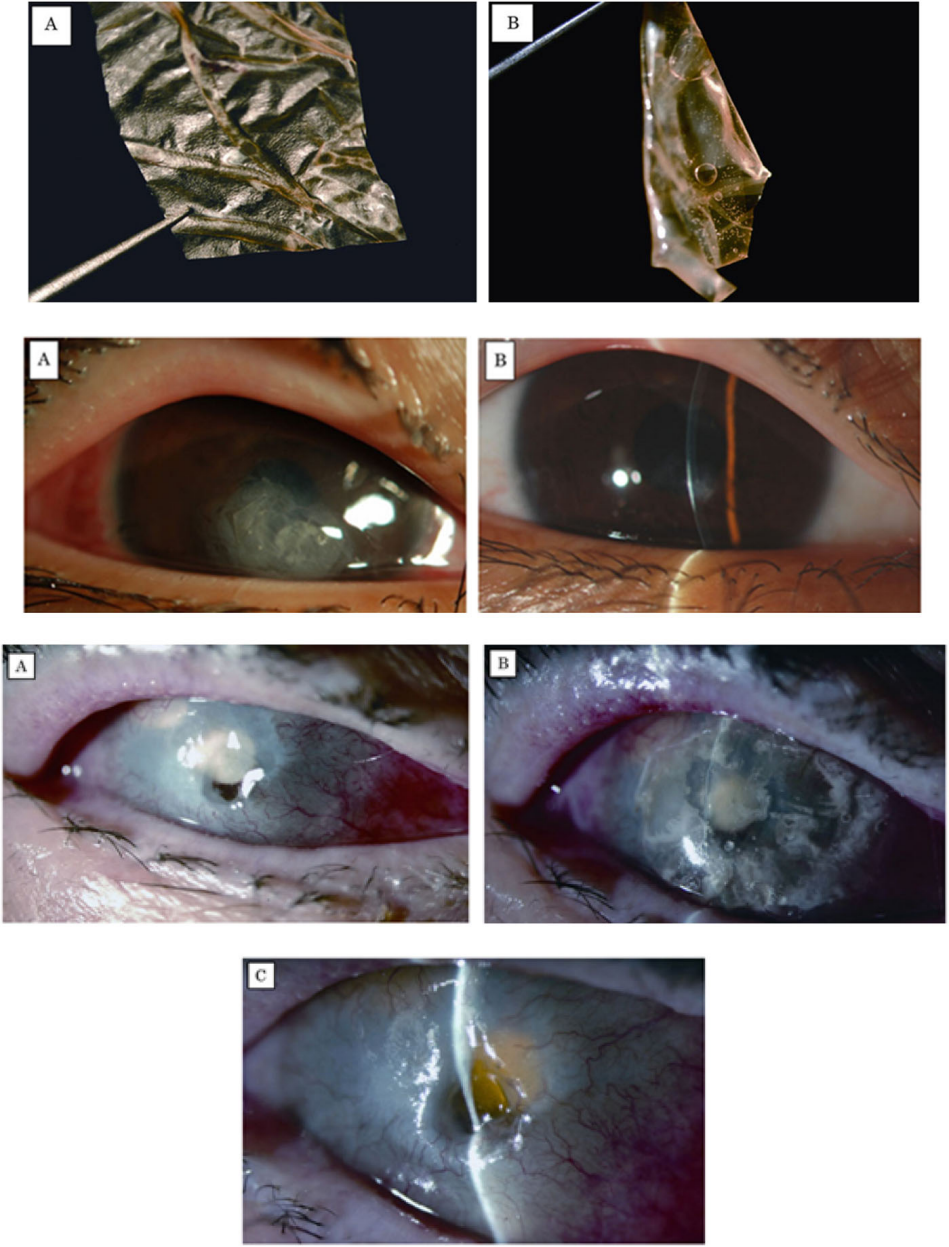
(top) Photographs of sterilized dry AM cross-link with GTA, (**A**): dry state, the AM can easily grasped with forceps, (**B**): hydration state it becomes flexible and similar to fresh AM; (middle) images of HDCL-AM patching in a 13-year-old boy with corneal perforation due to trauma. **A** Single-layer patch of HDCL-AM applied with tissue adhesive. **B** When the HDCL-AM patch fell off spontaneously 17 days after the treatment, faintly hazy repaired tissue of normal thickness was observed; (bottom) images of HDCL-AM patching in a patient with corneal perforation in a blind eye caused by secondary glaucoma. **A** Corneal stromal defect measuring 3 mm in diameter at the center of the cornea. The anterior chamber had disappeared and the lens surface was visible through the perforation site. **B** Single-layer patch of HDCL-AM applied with tissue adhesive (HDCL-AM overlay). The aqueous humor was in direct contact with the HDCL-AM, and there was no aqueous leakage. A hydrogel contact lens bandage was installed for support. **C** When the HDCL-AM patch was removed 4 weeks after the treatment, the repaired corneal tissue was almost clear, but the repaired tissue remained thin. However, there was no aqueous leakage [108]

The AM treated with low concentrations of cross-linker (i.e., <0.03 mmol GTA/mg AM) manifested the reasonable compatibility with the human corneal epithelial cells. The samples with the highest cross-linking may show alterations in the cell morphology and decrease cell viability [112]. To conclude, the GTA concentrations are vital for customizing the chemically modified AM properties during cross-linking treatment for use in limbic stem cell niche. Kitagawa et al. [108] reported using the hyper-dried, tissue adhesive cross-linked AM (HDCL-AM) patching as an initial therapy for the corneal perforation which is repaired in 17–28 days. During collagenase digestion experiment, the HDCL-AM did not dissolve in 48 h, whereas the cryopreserved AM completely dissolved within 60 min. The AM was cross-linked with GTA (0.1%) solution, washed carefully with saline and dried by a hyper-drying in which, the temperature within this method was controlled in range of 5–35 °C under vacuum conditions (Fig. 7(top, A) and (top, B)). Also, Images of HDCL-AM patching have shown in Fig. 7(middle) and 6 (top, A-C) [108].

#### Carbodiimide

**Fig. 8:**
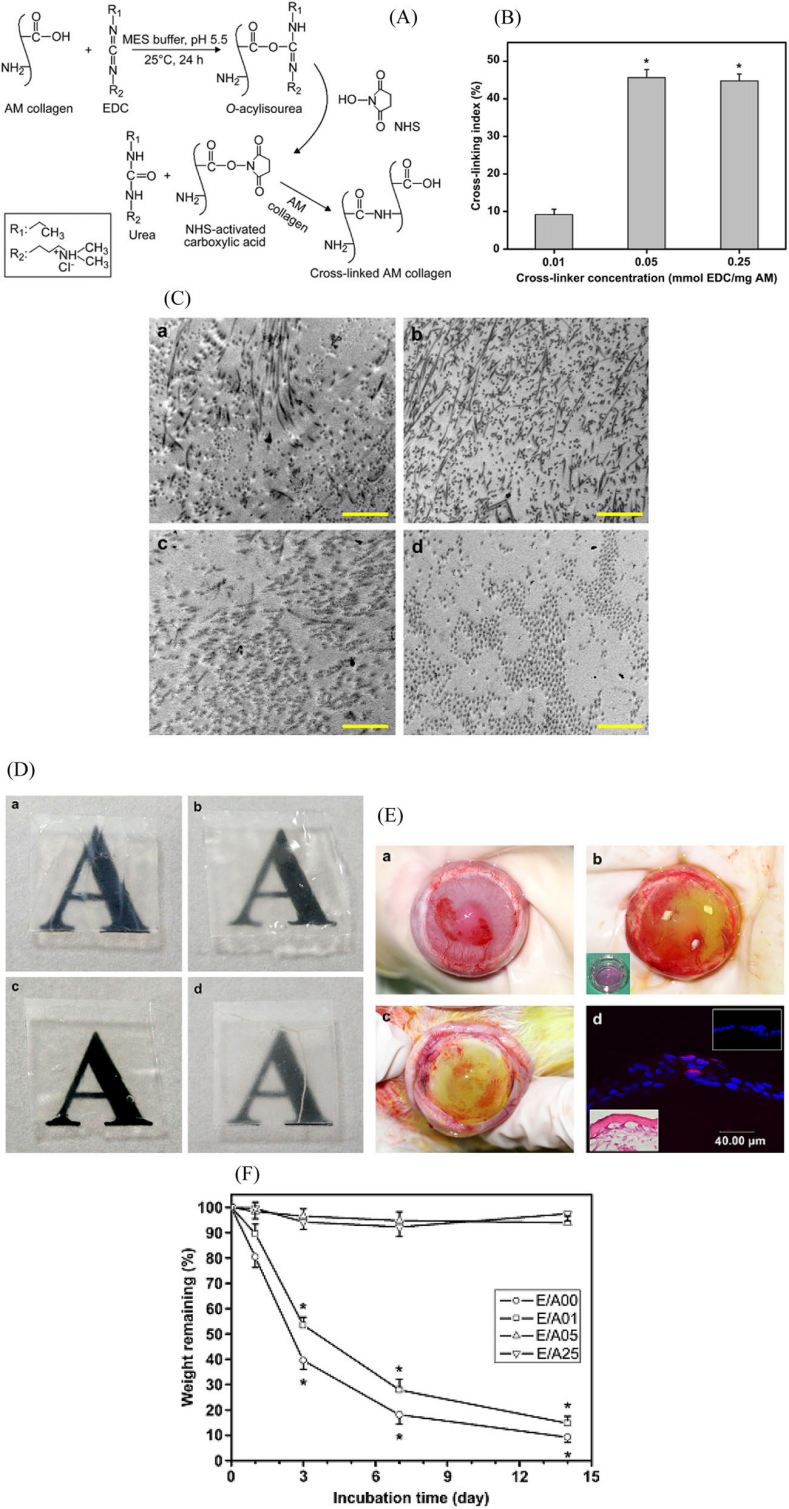
(**A**) Cross-linking reaction scheme of AM collagen with EDC; (**B**) cross-linking index of AM as a function of EDC concentration; (**C**) representative of the Transmission Electron Microscopic (TEM) images of various AM samples. (**a**) E/A00, (**b**) E/A01, (**c**) E/A05, and (**d**) E/A25 groups. Scale bars: 1mm; (**D**) typical macroscopic views of typescript beneath various AM samples. (**a**) E/A00, (**b**) E/A01, (**c**) E/A05, and (**d**) E/A25 groups; (**E**) reconstruction of the corneal surface by ex vivo expanded autologous rabbit limbal epithelial cells (LECs) cultured on EDC cross-linked denuded human AM; (**F**) the graph of weight remaining (%) versus incubation time (day) [153]

The AM modification by 1-ethyl-3-(3-dimethyl amino propyl) carbodiimide hydrochloride (EDC)/N-hydroxysuccinimide (NHS) does not insert outer structures into the biomaterial network and is thus considered a more biocompatible method. In a study by David Hui-Kang Ma et al. [31], the EDC/NHS for LEC carrier application reported (Fig. 8(A)). Figure 8(B) shows the increase in the cross-linking percentage with increasing concentration of the EDC. Results indicated that chemical cross-linking saturated at a concentration of 0.05 mmol EDC/mg AM. With the concentration of 0.05 mmol EDC/mg AM, cross-linker could remarkably increase optical transparency, collagenase digestion resistance and the thermal and mechanical stability. Continuous permeation of the Albumin via the cross-linked AM will lead to the cell growth on the matrix surface. Furthermore, the cross-linked samples with EDC could support LEC proliferation and maintaining the epithelial progenitor cells in vitro and in vivo (Fig. 8(C–F)). Corneal inflammation and neovascularization were induced after NaOH injury and surgical removal of limbus. Two weeks after cultivation, confluent culture of LECs (insert) was transplanted to the corneal surface, with limbal explants still remained on the epithelial sheet. One week after transplantation, there was further decrement in corneal inflammation and neovascularization, although some extravasated blood trapped under AM was still visible. The HE staining revealed the intact AM with overlying epithelium. The positive staining was also discovered in some basal and suprabasal epithelial cells [31].

**Fig. 9:**
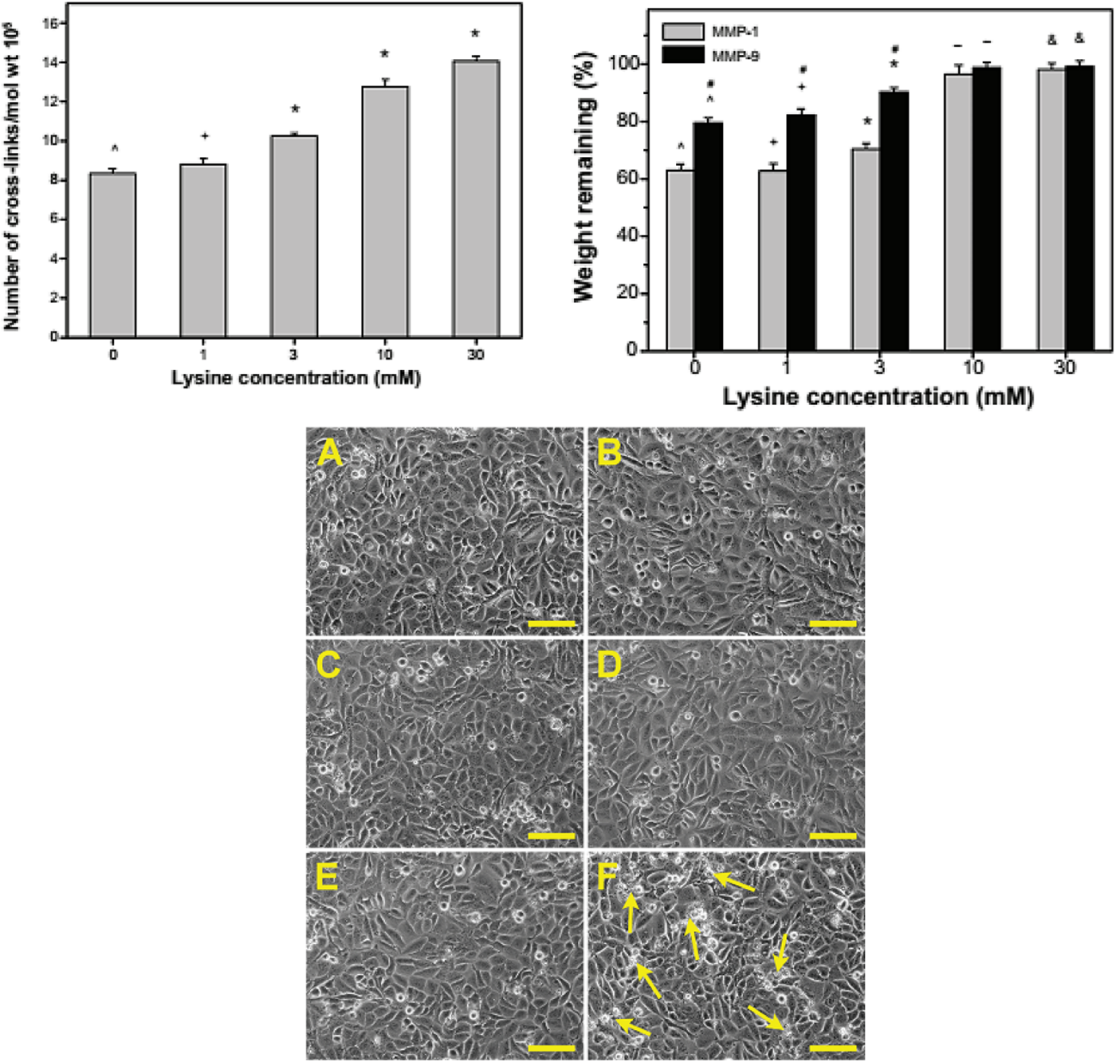
(top, left) Number of cross-links per unit mass as a function of l-lysine-pretreated concentration; (top, right) weight remaining in various amniotic membrane samples after incubation at 37 °C for 4 weeks (bottom); the phase-contrast micrographs of human corneal epithelial cell cultures. (bottom, **A**) controls (without materials) after a 3-day exposure to various amniotic membrane samples; (bottom, **B**) lys0, (bottom, **C**) lys1, (bottom, **D**) lys3, (bottom, **E**) lys10, and (bottom, **F**) lys30; dead cells (arrows) are presented in (bottom, **F**). scale bars: 50 μm [104]

The biocompatibility and stability are both prominent factors in studying the biomaterial cross-linking and its applications. Jui-Yang lai et al. [104] used l-lysine as an additional bridge of amino acid for investigating the cross-linked AM stabilization via EDC/NHS for application as a limbal stem cell niche. The residual amount of the amino acid is highly associated with l-lysine concentration which influence the hydrophilicity and structure of the scaffolding materials. The alteration in biological and thermal stability are in accord with the cross-links number per AM unit mass (Fig. 9) [104].

**Fig. 10:**
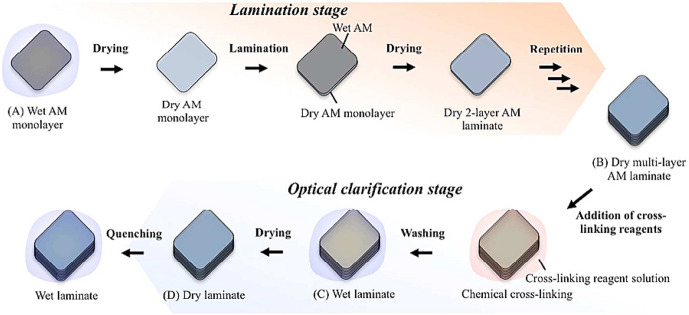
Production of cross-linked AM tissue laminates [119]

The transplantation technique for LECs cultivation on AM substrate may cause restoring vision for patients distressed with one-sided corneal stem cell deficiency [46, 131]. For AM grafts, peptide bridges are usually stabilizing collagen nanofibers. The corneal diseases can increase the tissue collagenase action and thereby accelerate the AM matrices degradation after surgery [101]. Jui-Yang lai et al. [31] cross-linked the AM with exogenous carbodiimide for improving it against enzymatic cleavage. For further forming various amide bonds between the amino and carboxyl groups of the AM collagen, the matrices exhibited an enhancement in resistance to the collagenase digestion, that probably resulted in the protection effect produced by aggregation of the collagen nanofibers. However, the cross-linking efficiency has been restiricted by treatment of the AM with carbodiimide, approximately 20% of the lost weight during the degradation within 2 to 4 weeks perceived for the carbodiimide-treated AM with a saturated cross-linking index equal to 45% and the remained tissues degrade gradually [107]. Figure 10 depicts a method developed by Hariya et al. [132], which indicates an innovative way for the fabrication of resilient and transparent cross-linked AM tissue covers. Carbodiimide was used for cross-linking and up to eight layers were collected to fabricate a rougher and optically clearer graft for corneal transplantation [79].

**Fig. 11:**
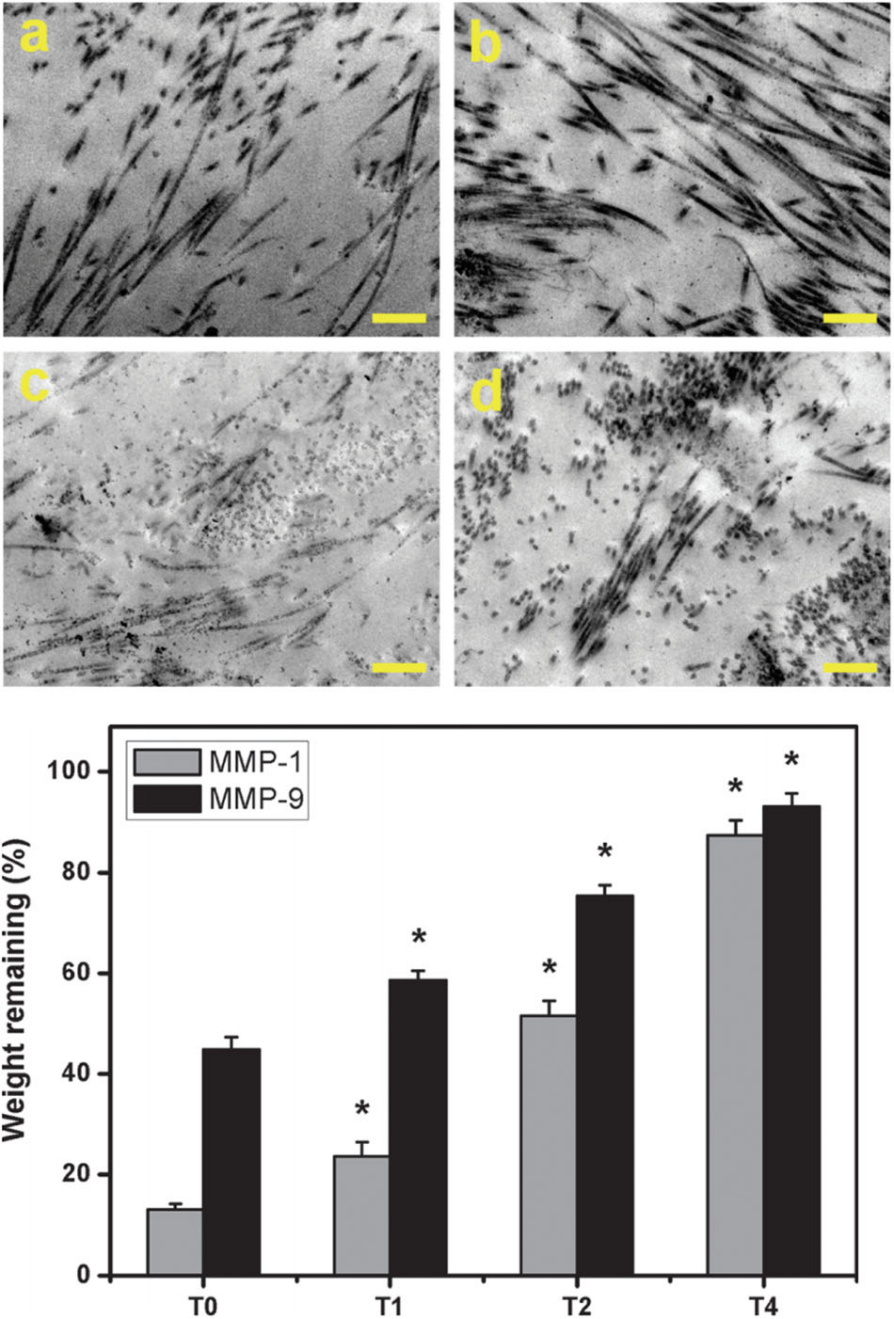
Representative transmission electron microscopic images of various AM samples(with various time cross-linke). (top, **a**) T0, (top, **b**) T1, (top, **c**) T2, and (top, **d**) T4 groups (T0: whitout cross-link, T1: cross-link within 1 h, T2: cross-link within 2 h, T4: cross-link within 4 h) Scale bars: 500 nm; (bottom) weight remaining of various AM samples after incubation at 37 C for 4 weeks in BSS containing MMP-1 or MMP-9(matrix metalloproteinase-1 (MMP-1, EC 3.4.24.7), MMP-9 (EC 3.4.24.35)) [105]

Jui-Yang Lai et al. [105] also investigated a limbal epithelial cell (LEC) AM scaffold cross-linked with the carbodiimide. By elevation of the treatment time, the cross-linked AM exhibited larger diameter of nanofiber and rougher texture. Noticeable increases in the water content, light transmittance and enzymatic degradation resistance probably because of the aggregation of collagen fibrils in biological tissues. All tested AM materials were nontoxic to cultured human corneal epithelial cells and maintained anti-inflammatory activity, indicating the safety and acceptability of carbodiimide. In addition, AM samples having higher cross-linking degree showed significantly increased LEC growth and enhanced p63 and ABCG2 gene expressions (Fig. 11) [105].

**Fig. 12:**
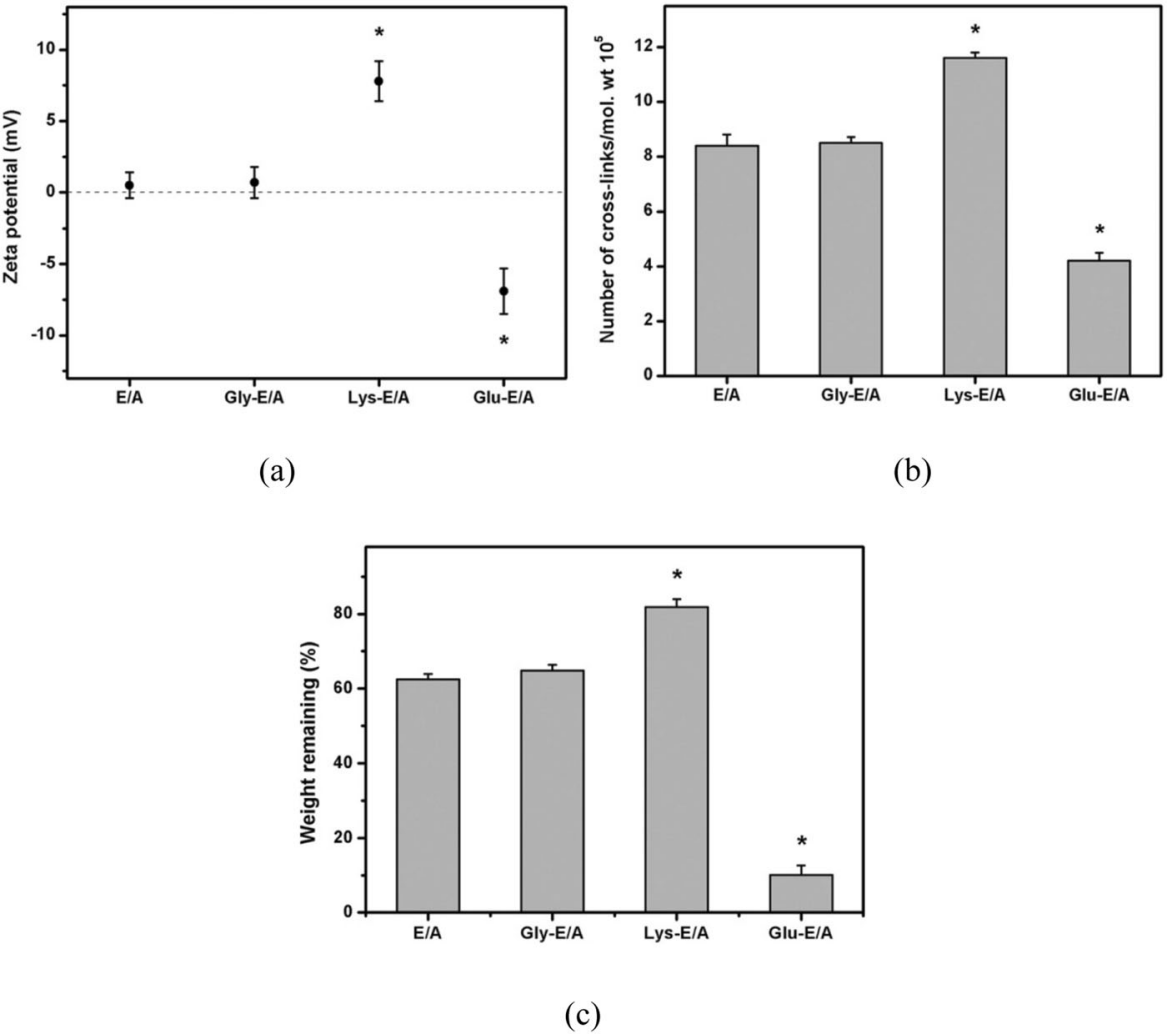
(**a**) Zeta potential of AM samples following carbodiimide-mediated cross-linking in the presence of different types of amino acids, (**b**) Number of cross-links per unit mass of AM samples following carbodiimide-mediated cross-linking in the presence of different types of amino acids, (**c**) Weight remaining of various AM samples after incubation at 37 °C for 4 weeks in BSS containing collagenase. An asterisk indicates statistically significant differences as compared to the E/A groups (i.e., carbodiimide cross-linking in the absence of amino acid bridges) [103]

The EDC/NHS cross-linked denuded AM (CLDAM) matrix, with its rougher ultrastructure and higher rigidity, well maintain the HLE progenitor cells in vitro, probably by actuating integrin b1/ILK, which indirectly actuated Wnt/bcatenin and later deltaNp63a. Crosstalk among the integrin b1/ILK and Wnt/b-catenin paths seemed to play a vital role in survival of the limbal progenitor cell on EBM [102]. Taghiabadi et al. [133] fabricated a spongy 3D scaffold from the acellular AM ECM. The ECM of amnion was composed of collagen I, collagen III, collagen IV and fibronectin. The HAM consulted the applicable wound protection and had a symbolic outcome on the pain decrease [23]. 3D natural and synthetic scaffolds also play a main role in maintenance of cell proliferation and tissue regeneration [134]. Interactions between the cells and ECM are responsible for the control of cell action. Hence, the cells grown in a 2D monolayer cannot cope with the relative complexity of in vivo micro-environment. The cells cultivated on 2D layer such as culture plates, lose numerous critical signals, important regulators, and tissue phenotypes. Cells growing in 3D have varied propagation capacity, extracellular matrix synthesis, cell congestion and metabolic functions [134]. Thanks to its unique features and similarity to the skin, it can be a good candidate for the skin tissue engineering. Lai et al. [103] investigated the AM cross-linked with carbodiimide in presence of the amino acid bridges. The AM tissues was treated with glutamic acid, lysine or glycine and cross-linked chemically to investigate the role of various amino acid types in processing the collagenous biomaterial. Based on of zeta potential data, the charge of membrane surface is affected whether using the positively, negatively charged and also uncharged amino acids. Water content angle and tensile strength measurements confirmed that addition of the lysine molecules can enhance the efficiency of cross-linking and degree of dehydration while the insertion of glutamic acid will reduce the cross-link numbers per unit mass of the modified collagen. The variances in the density of cross-linking determined the biological and thermal stability by DSC and degradation tests in vitro (Fig. 12) [103].

**Fig. 13:**
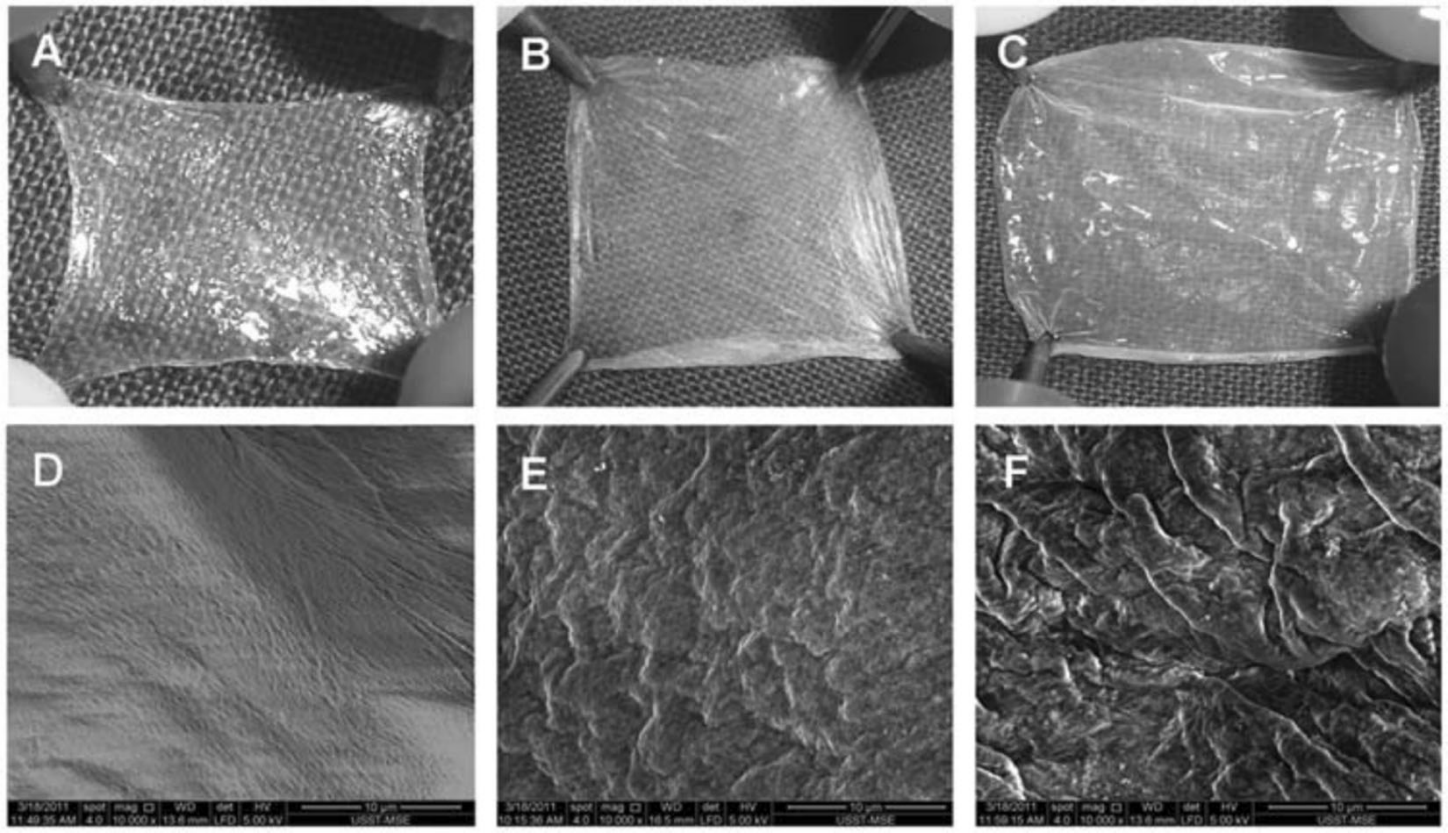
Changes over time in the gross appearance and SEM images of cross-linking DAM. Gross appearance and scanning electron microscope (SEM) images of 0 min-DAM (left panels), 5 min-DAM (middle panels), and 6h-DAM (right panels). As cross-linking time prolongs, the soft and smooth DAM gradually turns into a coiled and stiff one (**A–C**), and the uniformly reticular structure of the collagen fibers is replaced by fibrous cords formed by cross-linking (**D–F**) [135]

Huang et al. [135] reported the development of a dermal scaffold using AM having good biostability and intact basement membrane (BM) for quick transplantation and expansion of epidermal keratinocytes (EKs). For this target, decellularized amniotic membrane (DAM) was cross-linked via EDC for 5 min, 30 min, and 6 h (Fig. 13). By extending the time of cross-linking, the bio stability and mechanical strength of the DAM enhanced gradually, also enhanced its cytotoxicity against EKs. The cross-linked DAM for 5-min did not show a noteworthy cytotoxicity and had a good compatibility. It was grafted onto full-thickness skin defects in nude mice following the EKs culture and the cells endured well and made an epidermis similar to normal skin [135].

**Fig. 14:**
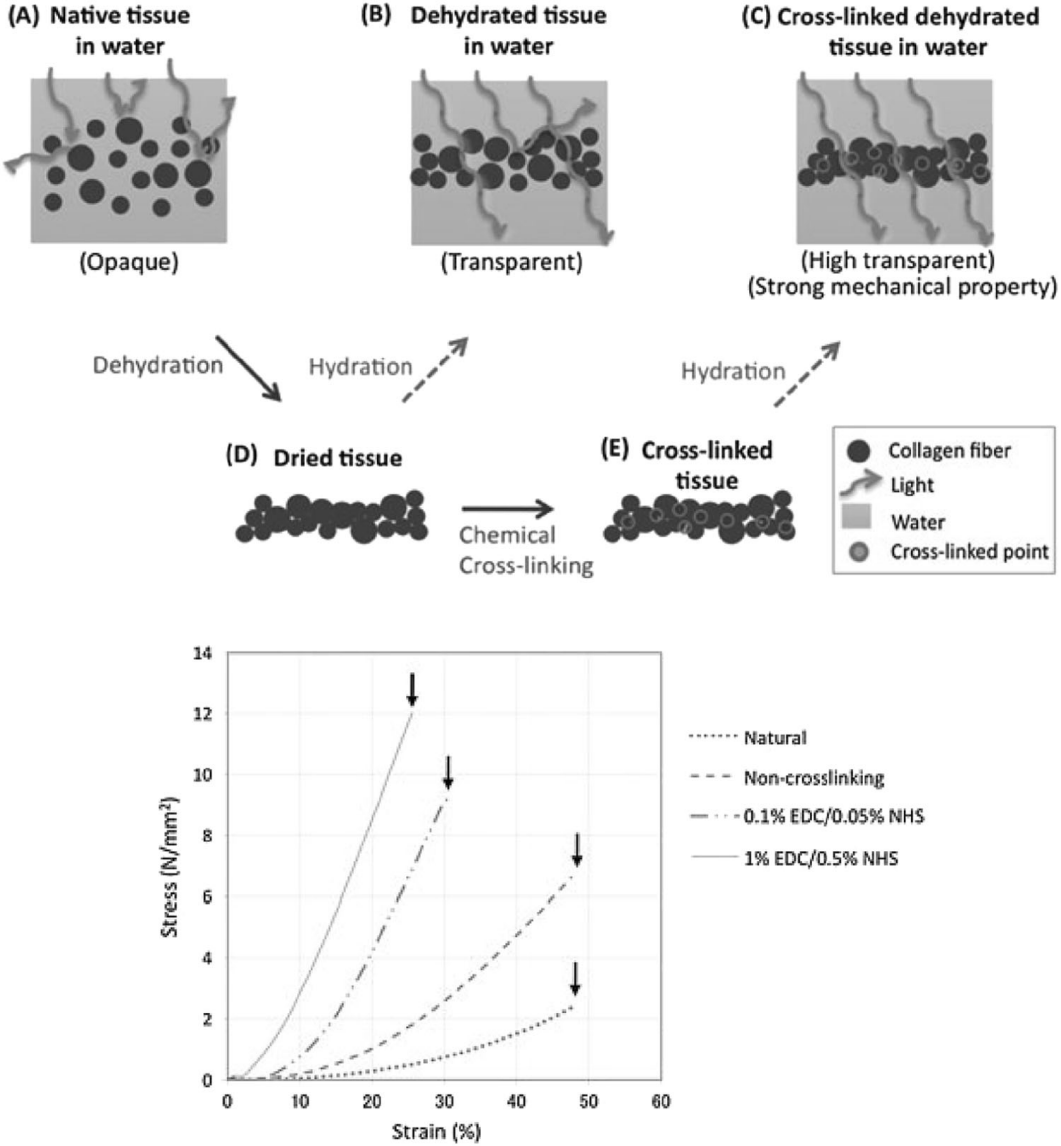
(top (**A** to **E**)) Proposed fiber structures in AM at each step during optical clearing alone by dehydration and two-step optical clearing by dehydration and cross-linking. Illustrations show the cross-sections of collagen fibers. Square backgrounds show collagen fibers in water. No background indicates that collagen fibers were held in air; (bottom (**F**)) stress-strain curves of wet human amniotic AM membranes treated by various processes including dehydration and cross-linking. Arrows represent the breaking point of each specimen.The dot line represents the stress-strain carve of native AM; the dashed line, no-cross-linked AM; the dash with two dots line, 0.1% EDC /0.05% NHS treated AM; the line, 1% EDC / 0.5% NHS treated AM [144]

Tanaka et al. [106] developed a technique for improving the mechanical and optical properties of the AM for ophthalmic delivery of the transparent implants in severe donor cornea deficiencies. For improving transparency of the AM, it was dehydrated gradually at 4–8 °C with and without chemical cross-linking. Then, dehydrated AM was cross-linked with EDC and NHS before rehydration. Figure 14(top) shows a schematic fiber structures of the AM during optical clearing alone by dehydration and by dehydration and cross-linking. After evaluation, Light transmittance improved from 50.9 to 77.7% at 550 nm. Even after rehydration with normal saline, light transmittance was partially higher indicating 70.1%. Remarkably, cross-linked AM showed a significantly greater light transmittance of 81.5% in wet conditions. Furthermore, after cross-linking, tensile strength was improved from 2.32 N/mm2 (native sample) to 11.78 N/mm2 (cross-linked samples) significantly (Fig. 14(bottom) and Table 4) [106]. Illustrations show the cross-sections of collagen fibers. Square backgrounds show the collagen fibers in water. No background indicates that the collagen fibers were held in air.

**Table 4:** Improved mechanical properties of wet human amniotic membranes treated with dehydration and cross-linking processes (means SD, *n* = 6) [106]

Low temperature dehydration	Cross-linking	Strain at break%	Force at break N	Tensile strength N/mm2	Young’s modulus N/mm2	Thickness µm
N/A	No	52.7 ± 12.6	1.37 ± 0.27	2.32 ± 0.38	0.046 ± 0.011	197 ± 15
Applied	No	54.0 ± 17.2	1.42 ± 0.34	4.50 ± 1.35	0.092 ± 0.040	108 ± 23
Applied	0.1% EDC/0.05% NHS	30.5 ± 7.9	2.34 ± 0.74	8.75 ± 2.55	0.280 ± 0.034	89 ± 10
Applied	1% DC/0.5% NHS	24.6 ± 6.17	1.99 ± 0.75	11.78 ± 6.23	0.467 ± 0.203	60 ± 10

#### Genipine

**Fig. 15:**
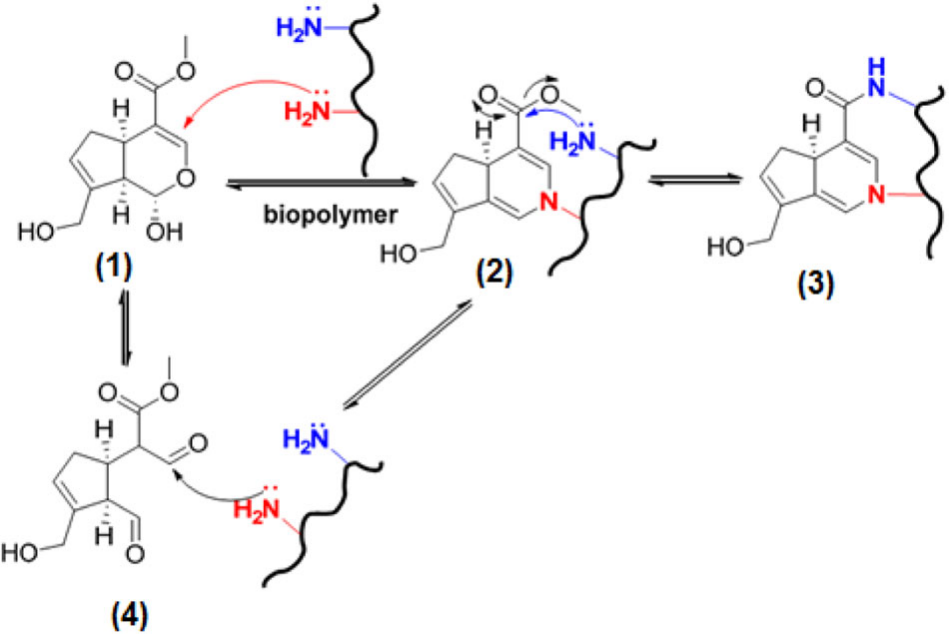
Mechanism of cross-linking for amine-containing biopolymers with genipine [136]

The mechanism of cross-linking for the genipine is not clear yet and different hypotheses have been proposed in this regard. Butler et al. [136] by investigating the reaction between the chitosan (biopolymer naturally containing primary amine groups), bovine serum albumin (BSA), and gelatin with genipine proposed a mechanism as depicted in Fig. 15.

The initial step was suggested to be the nucleophilic attack of one amine group from the biopolymer (in red) to α, β-insaturated ester, with the corresponding opening to yield 2, in a similar way as postulated by Touyama [137] (Fig. 15). In the second step, another amine group from the biopolymer will attack the methoxycarbonyl group to produce a secondary amidetype linkage, with the concomitant release of methanol, to reach the cross-linked compound 3. According to Di Tomasso et al. [138, 139], the first step would alternatively proceed via a previous opening of the hemiacetal 1 to the dialdehyde 4, which will be attacked by the amino group of the biopolymer to furnish 4 [140].

A research group prepared a genipin cross-linked HAM and investigated its characteristics which were important for its application as a scaffold, specifically swelling percentage, native extracellular matrix proteins retention, in vitro degradation, ultrastructure, mechanical strength, biocompatibility and optical clarity. For this purpose, the HAM divided into 3 groups: native (nAM), decellularized (dAM) and genipin cross-linked (clAM) groups. The dAM and clAM groups were decellularized by thermolysin (TL) and sodium hydroxide (NaOH) solutions. Following, clAM group was cross-linked with genipin (0.5% and 1.0% (w/v)). The degradation rate of the clAM was slowest and was still morphologically intact after incubation in collagenase type I (0.01%) solution within 30 days. when compared to nAM and 1.0% clAM, cell attachment on 0.5% clAM and dAM was higher. Briefly, in comparison to the dAM and nAM, clAM showed a better biocompatibility and biostability and clAM was appropriate as scaffold for different tissue engineering approaches [141].

**Fig. 16:**
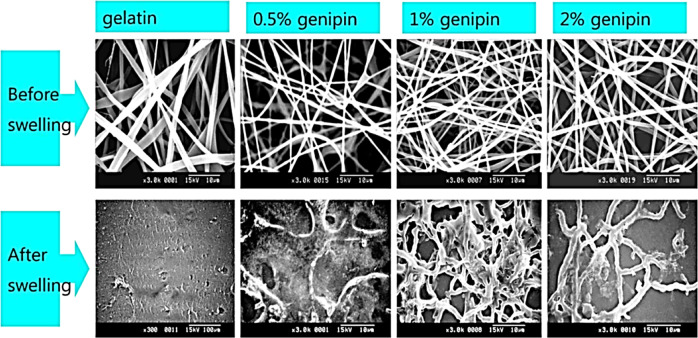
Scanning electron microscopy images of the cross-linked gelatin nanofibers and their swelling behavior with different concentrations of genipin [142]

Hyun et al. [142] also cross-linked the gelatin nanofibers with genipin. With increasing the genipin concentration from 0.5% (w/ v) to 2% (w/v), the swelling ratios reduced from 725% to 445% (Table 5). Figure 16 illustrated the scanning electron microscopy images of the cross-linked gelatin nanofibers and their swelling behavior with different concentrations of genipin. The cell culture results recommend that, gelatin nanofibers cross-linked with 0.5% (w/v) genipin promotes cell proliferation following increasing the cell culture time from 1 day to 5 days. In addition, cell viability for nanofibers increased with elongated cell culture time. However, the cell viability reduced by increasing concentrations of genipin [142].

**Table 5:** Characteristics of genipin cross-linked gelatin film [142]

Sample	Gelatin concentration (%(w/v))	Genipin concentration (%(w/v))	Swelling ratio (%)	Nano fiber diameters before swelling (nm)
1		0.5	725.14 ± 34	420 ± 120
2	20	1	584.71 ± 26	620 ± 160
3		2	445.31 ± 15	820 ± 180

**Fig. 17:**
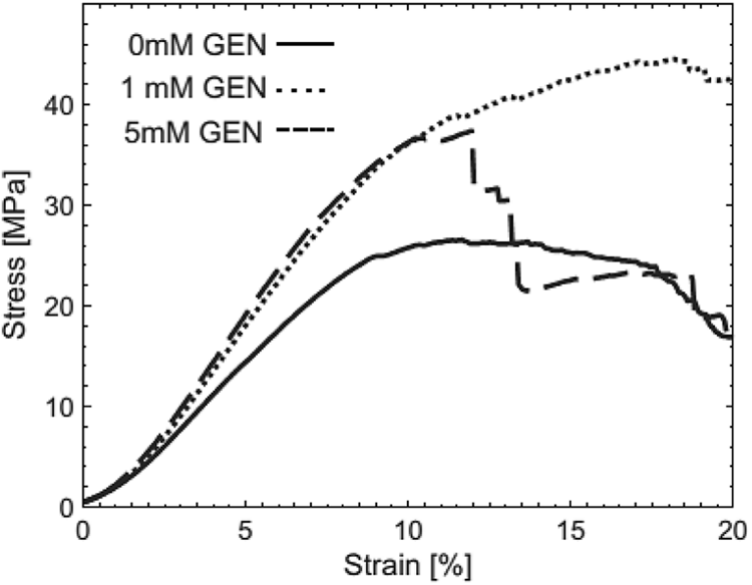
ample stress–strain curves from a triple of tendon strips cut from the same tendon [143]

Fessel et al. [143] reported that the genipin is proper for the mechanical improvement of the collagen tissues and also to evaluate the effects of genipin on tendon cells and their matrix. They established an in vitro dose-response baseline. According to Fig. 17, an enhancement in the mechanical properties can only be attained by accepting some degree of cytotoxicity, However, cell survival after treatment may be sufficient for tissue [143].

#### Aluminum sulfate

**Fig. 18:**
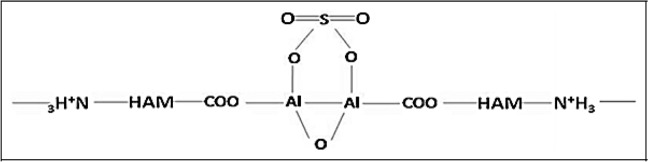
Coordination complex of Al_2_(SO_4_)_3_ with carboxyl groups of HAM [109]

Sekar et al. [109] cross-linked the HAM by Al_2_(SO_4_)_3_ (Fig. 18) and evaluated its mechanical properties. Corneal LECs was cultured in vitro to assess whether the cross-linked HAM support the attachment and proliferation or not. In the cross-linked HAM compared to HAM, about 125% increase in the tensile strength was observed. Infrared spectroscopy confirmed the cross-linking of HAM with Al_2_(SO_4_)_3_. The cross-linked HAM was sterile for up to 1 year, and tissue culture studies have shown its possibility to be used as a limbal transplant [109].

## Physical cross-linking

**Fig. 19:**
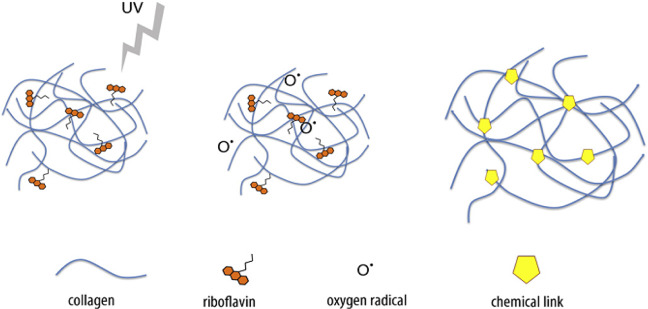
Photo-initiated free radical cross-linking reaction induced by riboflavin. Once irradiated with UV radiation an H + ion is released, producing a radical active oxygen 0. Collagen amine groups can then react generating covalent bonds [144]

Design of materials with the sutable mechanical properties is crucial and challenging for their effective application in tissue engineering. The collagen and riboflavin were utilized for the hydrogel design to mimic the soft tissues like liver. Collagen based hydrogels were obtained after a two-step gelation technique. Initially, a physical gelation step like change in temperature and pH was utilized to fix an exact shape; then, the stiffness in formed following photo-based cross-links. Cross-linking step was started via UV (ultra-violet) radiation to attain radical polymerization of the collagen chains by riboflavin (Fig. 19). Tirella et al. [144] showed that, between 0.9 and 3.6 kPa elastic modulus of collagen hydrogels can be adjusted by varying collagen concentration, UV irradiation in the presence of riboflavin and freeze-drying [144].

**Fig. 20:**
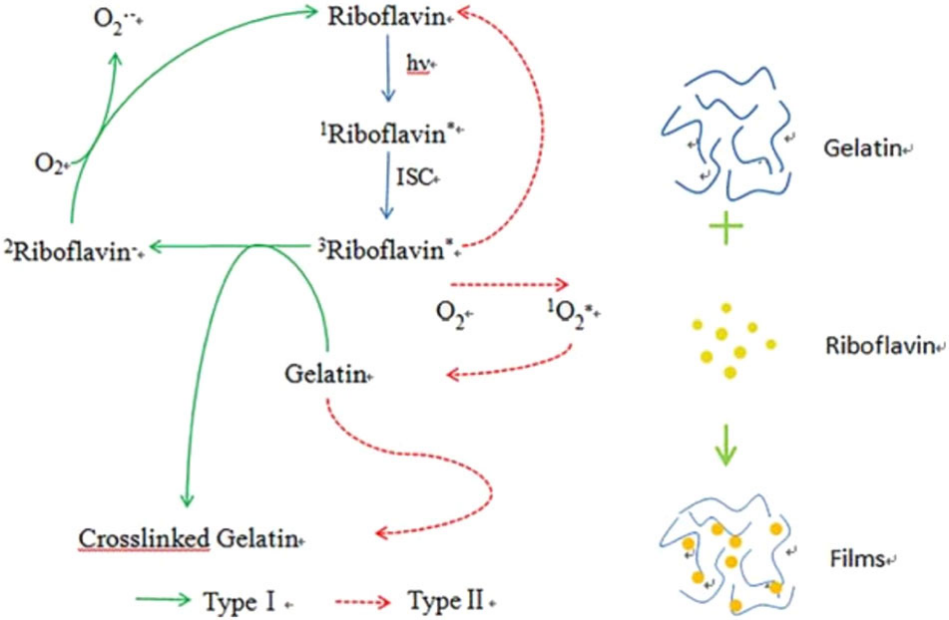
Rationale of UV-induced photocross-linking mediated by riboflavin through type I and type II and its enhancing on gelatin films [145]

In another researvh, Wang et al. [145] improved the characteristics of fish gelatin (FG) films by using riboflavin as a photosensitizer during UV irradiation cross-linking process (Fig. 20). The Young’s modulus and tensile strength of RmUV irradiated FG films were significantly higher than those of FG films in wet or dry conditions (Table 6). The ultraviolet/riboflavin irradiation is a potentially effective process for improving the performance of FG films [145].

**Table 6:** Influence of UV irradiation and riboflavin addition levels on mechanical properties of the resulting films under either “dry”or“wet” conditions [145]

Riboflavin (wt%)	Thickness (mm) Dry Wet		Young’s modulus (GPa) Dry Wet		Tensile strength (MPa) Dry Wet		Elongation at break (%) Dry Wet	
0	51 ± 3	121 ± 6	2436 ± 58	325 ± 29	21.50 ± 1.36	3.24 ± 0.24	9.51 ± 0.37	87.23 ± 4.23
0.01	52 ± 1	118 ± 4	2504 ± 89	345 ± 32	22.45 ± 1.89	3.32 ± 0.21	8.89 ± 0.45	83.25 ± 5.23
0.02	52 ± 3	115 ± 5	2678 ± 75	386 ± 35	23.56 ± 1.92	3.47 ± 0.28	8.58 ± 0.37	78.45 ± 4.21
0.05	54 ± 2	112 ± 4	2803 ± 48	438 ± 35	25.05 ± 1.61	4.05 ± 0.31	5.77 ± 0.33	72.46 ± 3.62
0.1	52 ± 2	95 ± 5	3425 ± 83	642 ± 43	31.69 ± 1.28	4.67 ± 0.25	4.11 ± 0.32	34.23 ± 3.98
0.2	53 ± 3	85 ± 6	4613 ± 85	896 ± 42	36.26 ± 1.35	6.36 ± 0.31	2.85 ± 0.23	21.12 ± 4.01
0.5	55 ± 2	80 ± 7	4802 ± 89	934 ± 38	37.73 ± 1.38	7.72 ± 0.21	1.45 ± 0.25	15.24 ± 2.83
#	55 ± 2	105 ± 6	2606 ± 65	415 ± 34	22.36 ± 1.58	3.62 ± 0.23	8.42 ± 0.26	75.21 ± 4.31

# indicates that FGfilm added with 0.5 wt% riboflavin without UV radiation. Values were given as mean6standard deviation. Value with the different superscript letters within the same column is significantly different [145].

Sisson et al. [146] examined several cross-linking methods: D, L-glyceraldehyde (g90%) (GC), glutaraldehyde vapor, reactive oxygen species generation via a plasma cleaner and genipin in 70% (v/v) ethanol/water solution. For cross-linking electrospun scaffolds, glutaraldehyde was used in the vapor phase at a concentration of 0.5% (w/w) for 19 h. For cross-linking the gelatin scaffolds, reactive oxygen species generated by a plasma cleaner/sterilizer were used to insert the fibers in a metal perforated box. Both genipin and D,L-glyceraldehyde were dissolved in 70% (v/v) ethanol/water. Since glutaraldehyde is toxic at high concentrations, they followed other cross-linking methods. One easy alternative was the use of reactive oxygen species from plasma cleaners. Glyceraldehyde and genipin were known as an acceptable alternatives agents for cross-linking due to their lack of toxicity and their resistance to dissolution in the cell culture medium at 37 °C [146]. Fujisato et al. [29] studied the effect of HAM cross-linking via radiation and chemical approaches on its biodegradation and physicochemical properties. For radiation cross-linking, they used electron beam and γ-ray while chemical cross-linking was done by glutaraldehyde (GA). The tensile strength and elongation at break of the AM reduced after enhancing the irradiation dose of the electron beam and γ-ray irradiation, while GA cross-linking had no influence on the tensile properties. The diffusion of the proteins through the AM was not affected by the GA concentration during cross-linking. The radiation cross-linking seems to be much more effective compared to the GA cross-linking in restoring the degradation, due to the low cross-link density. The GA cross-linked AM was slowly degraded because the GA concentration at cross-linking is increased. When the AM cross-linked with GA and implanted subcutaneously in mice, the tissue response was mild, same as the native non cross-linked membrane [29].

**Fig. 21:**
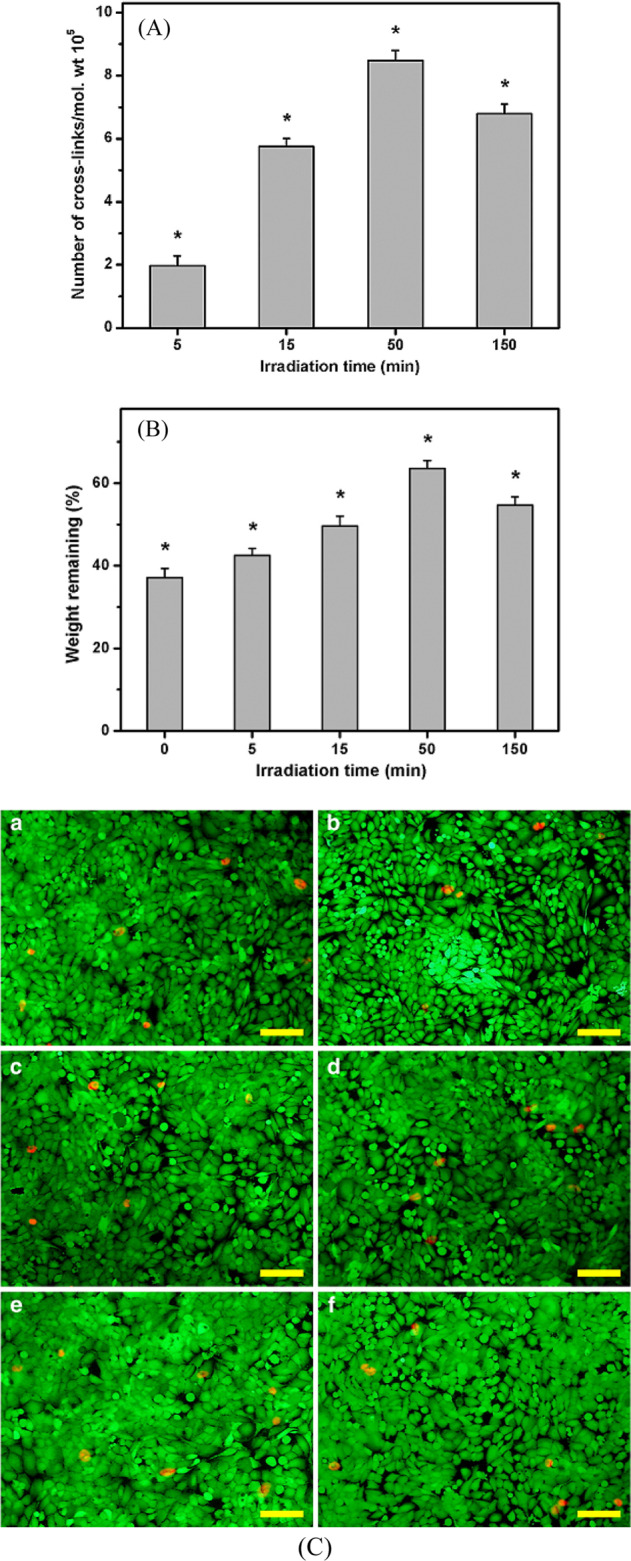
**A** Number of cross-links per unit mass of AM as a function of UV irradiation time; (**B**) weight remaining of various AM samples after incubation at 37 °C for 3 days in BSS containing collagenase; (**C**) the cell viability of HCE-2 cultures was determined by staining with Live/Dead Viability/Cytotoxicity kit in which live cellsfluoresce green and dead cellsfluoresce red. Fluorescence images of cells in (a) controls (without materials) after incubation for 3 days at 37 °C with extract medium conditioned with various AM samples (b) m0, (c) m5, (d) m15, (e) m50, and (f) m150. Scale bars: 50 μm [110]

The cardiac tissue engineering is a hopeful strategy for regenerative treatments to compensate the donor organs deficiency for transplantation as well contractile function. The stiffness and mechanical stability of the engineered tissue structures is crucial for the production of transplantable tissue alternatives to withstand the high pressure in the heart [147, 148]. While numerous collagen cross-linking methods have verified to be effective in stabilizing biomaterials, but it is not functional in cardiac tissue engineering since cell death happens in the treated area. It was represented a novel technique utilizing femtosecond (fs) laser pulses to improve the stiffness of collagen-based tissue scaffolds without damaging the cell viability. After a day of irradiation, measuring the stresses showed increased tissue stiffness up to 40% dependent on the amount of fibroblasts in the tissue. At that time, fluorescence imaging of cardiomyocyte mitochondrial activity confirmed that the cells remained viable and fully functional. Results showed that, collagen cross-linking induced by two photons has great potential for studying and developing artificially engineered tissues for reconstructive treatments [149]. Lai’s study demonstrated that the UV radiation physically cross-links the AM. The number of cross-links per unit of mass of photo cross-linked AM plays an important role in determining matrix permeability (Fig. 21(A)). Figure 21(B) reports that the biodegradability of these biological tissues strongly depended on the number of cross-linked structures, which was influenced by the duration of exposure to UV. The biologically cross-linked materials did not physically damage the corneal epithelial cells, regardless of the time of UV irradiation (Fig. 21(C)) [110].

**Fig. 22:**
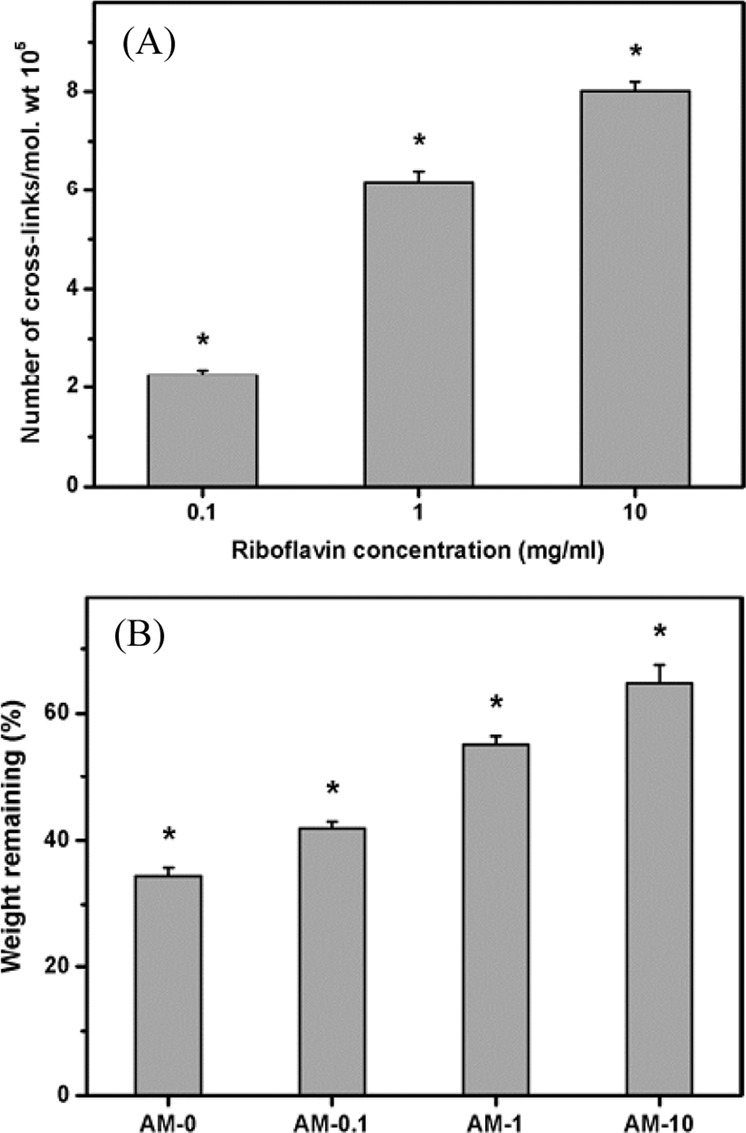
**A** Number of cross-links per unit mass of AM as a function of riboflavin concentration; (**B**) weight remaining of various AM samples after incubation at 37 °C for 3 days in BSS containing collagenase [150]

Lai et al. [150] investigated the effect of photoinitiator concentration on preparation of the cross-linked AM with photo on cultivation of the LECs. With increasing the riboflavin concentration from 0.1 to 10 mg/ml, the number of cross-links per unit mass of collagen matrix significantly increased (Fig. 22(A)). In addition, equilibrium’s water content, infrastructure, nanotopography and enzymatic degradability of AM samples are associated with cross-linked structure of UV-irradiated biological tissues (Fig. 22(B)). Regardless of the riboflavin concentration, the test specimens were completely biocompatible and retained anti-inflammatory activities, possibly due to the absence of exogenous cross-linked molecules in the protein matrices following cross-linking reaction. The LECs cultured on AM substrates with different cross-linking densities had different levels of enhanced stemness. The riboflavin concentrations may play an important role in modulating the properties of photo cross-linked AM as a new carrier of LEC [150].

Zhu et al. [145] made the cross-linking via activation of green light of Rose Bengal (RGX) and evaluated its possible damaging effects of the green light on retina and iris. The RGX enlarged corneal stiffness 1.9-fold after 1 day and 2.8-fold after 28 days compared with controls (Table 7). Keratocytes decreased only in the anterior stroma after 1 day and improved by day 28. Iris cells were not damaged thermally. No evidence of BRB degradation was observed after 1 or 28 days. The RGX-treated retina of RPE cells seemed normal, with intact cells protected by melanosomes, morphological receptor-derived outer portions, normal thickness of the outer core layer, and choriocapillaris containing intact corpuscles [151].

**Table 7:** Effect of RGX on corneal stiffness and thickness [151]

Treatment	Day	N	Stiffness, N/mm*	Young Modulus, N/mm2 *	Corneal Thickness, mm*
Control 1	1	3	1.25 ± 0.21	6.33 ± 1.38	0.507 ± 0.061
RGX, 150 J/cm2	1	3	2.38 ± 0.59^†^	10.9 ± 3.37	0.557 ± 0.074
Control 28	28	6	1.70 ± 0.47	11.2 ± 4.85	0.383 ± 0.026
RGX, 150 J/cm2	28	6	4.95 ± 1.86^‡^	29.7 ± 13.7^‡^	0.438 ± 0.095

*Mean6SD.

^†^P, 0.05 compared with control group.

^‡^P, 0.01 compared with control group

The UVA is also amply used in corneal collagen cross-linking. However, the cross-linking in sclera remained only in the experimental phase. The main limitation of UVA is its ability to penetrate into the sclera [152]. Theoretically, due to the negative relationship between the wavelength and the penetrating depth in the tissue, the blue light in the sclera is able to penetrate better. In addition, compared to 365 nm, the wavelength is longer and the probability of biological damage is lower [153]. Iseli et al. [154] performed Blue light (465 nm at 26 mW/ cm2) for 20 min by cross-linking on 6 scleral rabbits [155]. Four weeks later, the measurement of stress-strain caused a threefold increase of sclera stiffness by scleral cross-linking compared to the uncontrolled eye [155].

## Composite materials

**Fig. 23:**
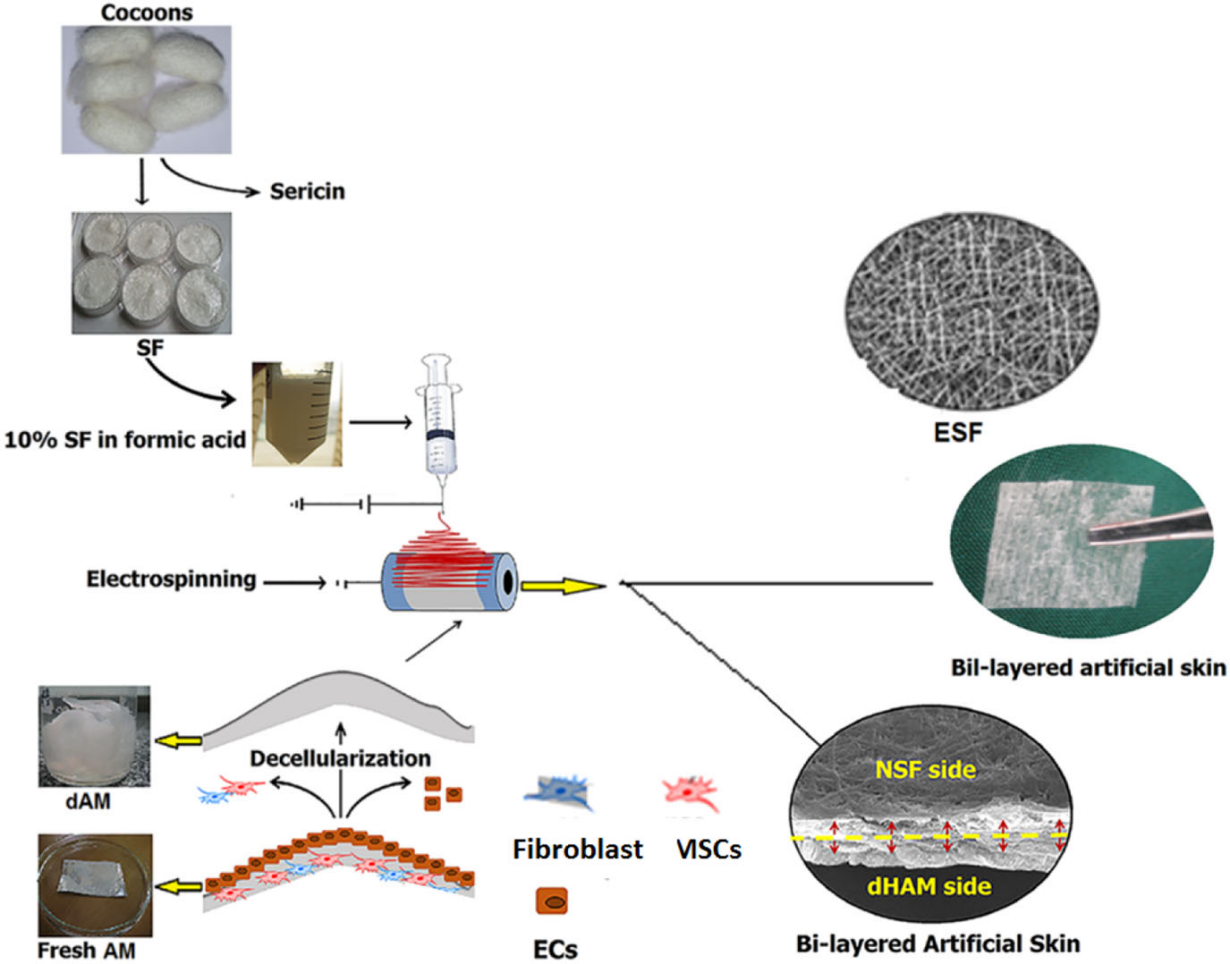
Schematic preparation of the bi-layer artificial skin preparation. dAM decellularised human amniotic membrane, ECs epithelial cells, AM human amniotic membrane, MSCs mesenchymal stem cells, ESF nanofibrous silk fibroin, SF silk fibroin [91]

The composite scaffolding has been also developed as an alternative way to adjust the properties of the AM. Gholipourmalekabadi et al. [91] developed a 3D bi-layer scaffold from decellularized AM with viscoelastic silk fibroin (SF) nanofiber (Fig. 23). Prepared AM/SF 3D bi-layer scaffolds were poured into the ethanol to persuade β-sheet transformation as well as to obtain a highly coated and inseparable bilayer. The biological and biomechanical properties of the AM/SF scaffold were investigated. The results indicated a significant better mechanical property of the AM/SF compared to the control AM. Both AM and AM/SF have suitable adhesion cell types without cytotoxicity against the Adipose. The AM/SF scaffold with autologous ATMSCs had excellent proliferation and cell adhesion accompanied by the production of growth factors which aids as a possible use in clinical settings in skin renewal [91].

**Fig. 24:**
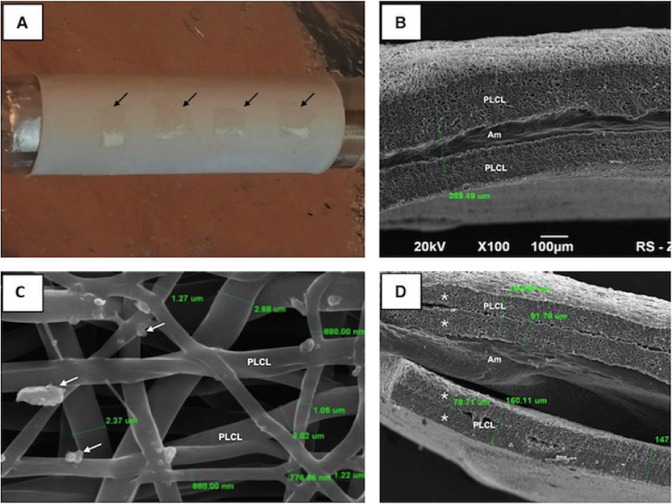
Preparation and structure of biocomposite. **A** The pieces of AM (black arrows) placed onto a sheet of PLCL nanofibers. SEM images are displayed in (**B**–**D**). **B** A cross-section image of the biocomposite material. The biocomposite material is 389 um thick with an inner cavity containing the AM. **C** Visible drops of glycerin used for AM preservation are observed on surface of PLCL nanofibers (white arrows). **D** Two pieces of delaminated biocomposite material. The borders between consecutive sheets of nanofibers (*) are clearly visible with AM inside [83]

Recent reports defined applications of the HAM for tissue engineering through its coating with the poly(lactic-*co*-glycolic acid) (PLGA) [156]. For developing the engineered blood vessels, the AM was mixed with fibrin and for tendon tissue engineering and it was combined with collagen glycosaminoglycan scaffolds [89, 90]. Adamowicz et al. [83] used the composite material method and denuded AM layered with electrospun poly(lactide-*co*-ε-caprolactone) (PLCL) for tissue engineering in urology. These composite scaffolds, which are illustrated in Fig. 24, were successfully implanted in rats [83].

Several composite and preparation methods have been used to supplement the AM for tissue engineering targets [157]. To produce a 3D bilayer of artificial skin, the denuded AM was collected separately with silk fibroin nanofibers [91]. Both sides of decellularized AM were coated with polyurethane to improve its mechanical strength and produce a surgical biocompatible network [158]. Another popular preparation method combined several AM scaffolds to produce the multilayer structures such as AM tissue laminations, the multilayer structures for the fabrication of tissue engineered blood vessels [29, 159, 160]. The surgical patches reported for use in ophthalmology [132, 161–163] and the oral and maxillofacial surgery [164, 165] skin defects have also been treated with HAM dissolved with hyaluronic acid hydrogel [166]. Finally, micronized HAM along with epidermal stem cells successfully repaired the full-thickness skin defects during transplantation in nude mice. The decellularized AM was coated with polyester urethane on both sides to improve its mechanical strength and produce a biocompatible surgical mesh [158]. Another popular preparation method is the combination of several hAM scaffolds to produce multilayer constructs such as the HAM tissue laminates. Multilayer structures have been reported for the production of engineered blood vessels [89, 159, 160], applications in ophthalmology [132, 162, 163], oral and maxillofacial surgery [83, 90], surgical patches [83] and Skin defects have also been cured with solubilized AM jointed with hyaluronic acid hydrogel [166]. Moreover, AM combined with epidermal stem cells successfully restored full-thickness skin defects when transplanted in nude mice [102, 167].

**Fig. 25:**
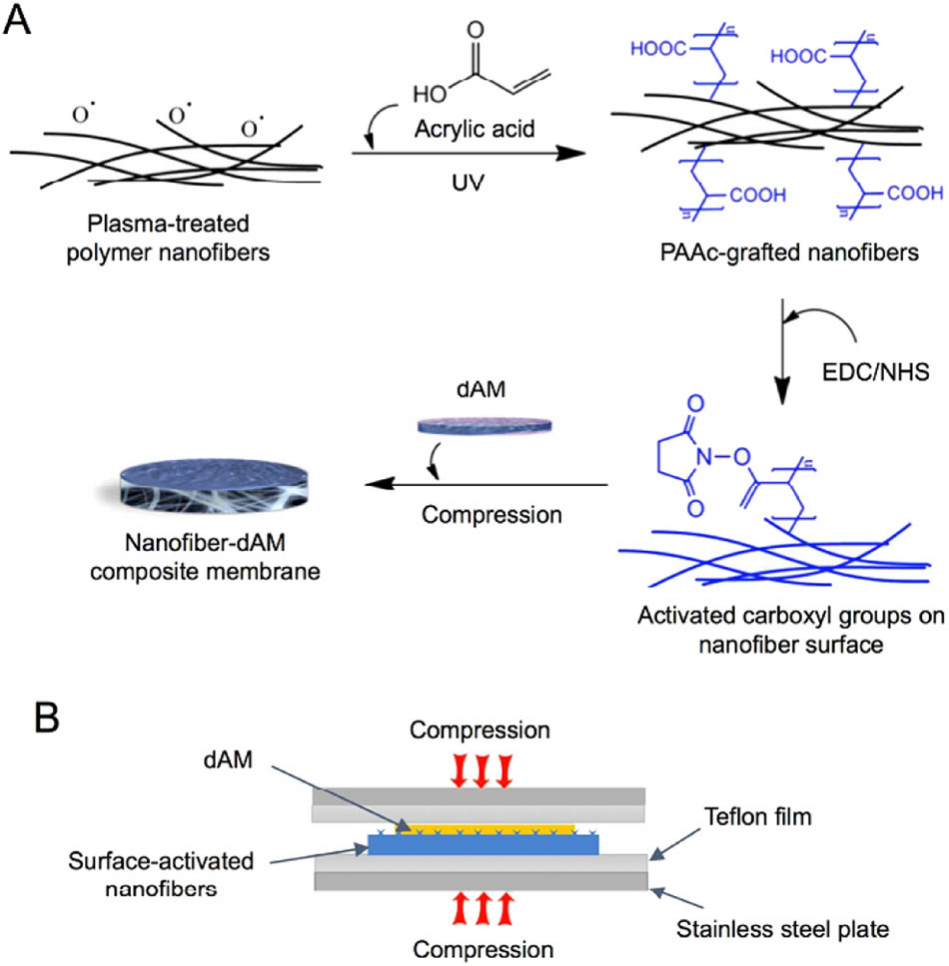
**A** Schematic of PAAc-grafting on electrospun nanofiber mesh, carboxyl group activation, and conjugation of activated fibers with dAM, forming an integrated bilayer composite membrane. **B** Illustration of mesh compression during interfacial bonding. PAAc poly(acrylic acid), NHS N-hydroxysuccinimide, EDC N-(3-dimethylaminopropyl)-N-ethylcarbodiimide [172]

Liu et al. [168] manifested that the polymer nanofibers have a morphological indications in supporting the stem cell expansion, differentiation and migration [168]. For instance, an electrospun PCL nanofiber has an ECM-mimicking structure with good flexibility and promising mechanical properties to support cell growth and adhesion [169, 170]. The polymer fibers can be functionalized to control present related bioactive moieties and cell adhesion [171]. A bioabsorbable polymer nanofiber and a decellularized AM can form a composite membrane with presenting interfacial bonding between the functional groups on the nanofiber surface and the protein components in the decellularized AM. The nanofiber plays the role as a strengthening layer providing mechanical support to the decellularized AM, while decellularized AM act as a biochemical support like the basement membrane structure for cell adhesion, growth, and survival. Figure 25 illustrates the method of making a bilayer, bonded nanofiber decellularized AM composite membrane and characterize the physical and functional properties of the developed membrane to support rabbit LSC growth, attachment, maintenance and report anti-inflammatory ability of the composite membrane and its ability to control macrophage phenotype [172].

## Conclusions and outlooks

The AM as a biological tissue material with the low immunogenicity has been extensively adopted in the clinical practices and treatments for a variety of ocular surface diseases such as the thermal or chemical burns, ocular cicatricial pemphigoid, pterygium, corneal ulcers and Stevens-Johnson’s syndrome. In addition to traditional applications as a surgical graft, the AM is an excellent candidate for the corneal tissue engineering and regenerative medicine. However, the application of AM as a scaffold is limited by its low biomechanical strength and rapid biodegradation. This work reviewed the cross-linking methods for improving the AM properties. In each section, the advantages and disadvantages of each method and clinical procedure results were outlined, and thereby it could be in part helpful for the future works.
